# Subtype-specific differentiation of cardiac pacemaker cell clusters from human induced pluripotent stem cells

**DOI:** 10.1186/s13287-017-0681-4

**Published:** 2017-10-16

**Authors:** Patrick A. Schweizer, Fabrice F. Darche, Nina D. Ullrich, Pascal Geschwill, Boris Greber, Rasmus Rivinius, Claudia Seyler, Karin Müller-Decker, Andreas Draguhn, Jochen Utikal, Michael Koenen, Hugo A. Katus, Dierk Thomas

**Affiliations:** 1 Department of Cardiology, Medical University Hospital Heidelberg, INF 410, D-69120 Heidelberg, Germany; 2 DZHK (German Centre for Cardiovascular Research), partner site Heidelberg/Mannheim, University of Heidelberg, INF 410, D-69120 Heidelberg, Germany; 3 Institute of Physiology and Pathophysiology, Division of Cardiovascular Physiology, Heidelberg University, INF 326, D-69120 Heidelberg, Germany; 4 Institute of Physiology and Pathophysiology, Division of Neuro- and Sensory Physiology, Heidelberg University, INF 326, D-69120 Heidelberg, Germany; 5 Department of Cell and Developmental Biology, Max-Planck-Institute for Molecular Biomedicine, Röntgenstrasse, 20, D-48149 Münster, Germany; 6 Unit Tumor Models, German Cancer Research Center (DKFZ), Heidelberg, INF 280, D-69120 Heidelberg, Germany; 7 Dermato-Oncology (G300), German Cancer Research Center (DKFZ), Heidelberg, INF 280, D-69120 Heidelberg, Germany; 8 Department of Dermatology, Venereology and Allergology, University Medical Center Mannheim, Ruprecht-Karl University of Heidelberg, Theodor-Kutzer-Ufer 1-3, D-68167 Mannheim, Germany; 9 Department of Molecular Neurobiology, Max-Planck-Institute for Medical Research, Jahnstrasse 29, D-69120 Heidelberg, Germany

**Keywords:** Pacemaker, Pluripotent stem cells, Automaticity, Differentiation

## Abstract

**Background:**

Human induced pluripotent stem cells (hiPSC) harbor the potential to differentiate into diverse cardiac cell types. Previous experimental efforts were primarily directed at the generation of hiPSC-derived cells with ventricular cardiomyocyte characteristics. Aiming at a straightforward approach for pacemaker cell modeling and replacement, we sought to selectively differentiate cells with nodal-type properties.

**Methods:**

hiPSC were differentiated into spontaneously beating clusters by co-culturing with visceral endoderm-like cells in a serum-free medium. Subsequent culturing in a specified fetal bovine serum (FBS)-enriched cell medium produced a pacemaker-type phenotype that was studied in detail using quantitative real-time polymerase chain reaction (qRT-PCR), immunocytochemistry, and patch-clamp electrophysiology. Further investigations comprised pharmacological stimulations and co-culturing with neonatal cardiomyocytes.

**Results:**

hiPSC co-cultured in a serum-free medium with the visceral endoderm-like cell line END-2 produced spontaneously beating clusters after 10–12 days of culture. The pacemaker-specific genes *HCN4*, *TBX3*, and *TBX18* were abundantly expressed at this early developmental stage, while levels of sarcomeric gene products remained low. We observed that working-type cardiomyogenic differentiation can be suppressed by transfer of early clusters into a FBS-enriched cell medium immediately after beating onset. After 6 weeks under these conditions, sinoatrial node (SAN) hallmark genes remained at high levels, while working-type myocardial transcripts (*NKX2.5*, *TBX5*) were low. Clusters were characterized by regular activity and robust beating rates (70–90 beats/min) and were triggered by spontaneous Ca^2+^ transients recapitulating calcium clock properties of genuine pacemaker cells. They were responsive to adrenergic/cholinergic stimulation and able to pace neonatal rat ventricular myocytes in co-culture experiments. Action potential (AP) measurements of cells individualized from clusters exhibited nodal-type (63.4%) and atrial-type (36.6%) AP morphologies, while ventricular AP configurations were not observed.

**Conclusion:**

We provide a novel culture media-based, transgene-free approach for targeted generation of hiPSC-derived pacemaker-type cells that grow in clusters and offer the potential for disease modeling, drug testing, and individualized cell-based replacement therapy of the SAN.

**Electronic supplementary material:**

The online version of this article (doi:10.1186/s13287-017-0681-4) contains supplementary material, which is available to authorized users.

## Background

The technology to reprogram human somatic cells back to pluripotency allows the production of patient-specific human induced pluripotent stem cells (hiPSC) and holds great translational promise [1]. In the field of cardiology, hiPSC-derived cardiomyocytes enable new approaches to studying cellular mechanisms of inheritable diseases and provide in vitro platforms for drug discovery and for regenerative therapies [2].

Differentiation of hiPSC or human embryonic stem cells (hESC) into cardiomyocytes can be achieved by various approaches using embryoid bodies or direct differentiation strategies [3–6], and yield a mixed population of cells including nodal-type and working-type (i.e., atrial and ventricular chamber) action potential (AP) properties [7]. Most culture media-based preparations were designed to enrich ventricular-type myocytes [8, 9], while approaches for targeted differentiation of pacemaker-type cells from human pluripotent cells are sparse and require sophisticated protocols [10]. However, enrichment of nodal cells in culture is an important strategy with respect to novel approaches for the treatment of bradycardias [11, 12]. Loss or dysfunction of sinoatrial nodal cells results in sick sinus syndrome (SSS), a collective term for disorders associated with failure in rate initiation or conduction from the sinoatrial node (SAN) to the atrium, comprising sinus bradycardia, SAN block, and bradycardia-tachycardia syndrome. In many cases, symptomatic SSS requires the implantation of an electronic pacemaker, which currently constitutes the only effective therapy for the syndrome [13]. With respect to limitations of electronic devices such as recurrent surgery due to limited battery durability, technical failure of implanted systems, and lack of responsiveness to the autonomic modulatory system, cell-based biological pacemakers may overcome these drawbacks [14, 15]. Considering the notion that cardiac differentiation of pluripotent stem cells yields mixed cardiac cell types [6, 7] and early, fetal cardiomyocytes comprise both pacemaker- and working-type properties [7], lineages were suggested to share a common developmental precursor [16–19]. Lineage separation depends on region-specific activation of gene programs [20] to yield either working-type myocytes that primarily develop myogenic characteristics, or nodal-type myocytes that express unique channel and receptor properties allowing for spontaneous depolarization and rate modulation according to neurohumoral demands.

We hypothesized that nodal type-specific differentiation can be controlled by media-based intervention in the dish to induce lineage specification without the necessity of genetic manipulation. According to this assumption, we observed that immature cardiomyogenic hiPSC-derived colonies, generated in a serum-free medium conditioned by visceral endoderm-like (END-2) cells, provide early nodal-type features. Timed transfer to a fetal bovine serum (FBS)-enriched medium drives further development to a distinct pacemaker-type phenotype resembling SAN-like characteristics.

## Methods

### Tissue procurement

All experiments involving human material were carried out in concordance with the Declaration of Helsinki. Written informed consent was obtained from all our patients. The study protocol involving human tissue samples for generation and differentiation of patient-derived induced pluripotent stem cells was approved by the ethics committee of the University of Heidelberg, Germany (#2009-350 N-MA). hiPSC lines #1 and #2 were derived from commercially available dermal fibroblasts (BioCat GmbH, Heidelberg, Germany, #DF-F-ZB; and ATCC, Teddington, UK, #CRL-2097, respectively); #3 was derived from a healthy patient donor, as described in Additional file 1.

All experiments involving rat and mouse tissue samples were carried out in accordance with the Guide for the Care and Use of Laboratory Animals published by the US National Institute of Health (NIH publication number 85–23, revised 1996) and the European Community guidelines for the use of experimental animals. Protocols were approved by the local regulatory authority (#T-38/14 Regierungspräsidium Karlsruhe, Germany). Mouse embryonic fibroblasts (MEF) were commercially obtained from BioCat GmbH (#CBA-310-CB).

### Generation and differentiation of hiPSC

hiPSC were generated from human fibroblast cultures obtained from skin biopsies of healthy control probands. Reprogramming was achieved using infection with single lentiviral particles carrying Oct-4, Sox2, Klf4, and cMyc (line #1). Additional hiPSC lines were generated in different laboratories with retroviral infection and three factors (Oct-4, Sox2, Klf4; line #2), or with a polycistronic lentiviral construct encoding four pluripotency factors (Oct-4, Sox2, Klf4, and cMyc; line #3). For details please refer to Additional file 1. hiPSC colonies formed teratomas and differentiated into all three germ layers when injected into immunodeficient mice (Additional file 2). To induce cardiomyogenic differentiation, hiPSC were co-cultured on an END-2 cell layer (treated for 3 h with 10 mM mitomycin C solution; Sigma-Aldrich, St. Louis, MO, USA) using knockout Dulbecco’s modified Eagle’s medium (knockout-DMEM; Thermo Fisher Scientific, Inc., Waltham, MA, USA) with 100 U/ml penicillin (Thermo Fisher Scientific), 100 μg/ml streptomycin (Thermo Fisher Scientific), 100× nonessential amino acids (Sigma-Aldrich), 0.1 mM 2-mercaptoethanol (Sigma-Aldrich), 100 μM ascorbic acid (Sigma-Aldrich), and 5 μM SB 203580 (Sigma-Aldrich), and was supplemented with 10 μM Y-27632 dihydrochloride (Abcam, Cambridge, UK). After 10–12 days, spontaneously beating clusters were observed. Beating clusters were mechanically isolated under light microscopy and transferred to standard cell medium containing Medium 199 (Sigma-Aldrich), 10% FBS (Thermo Fisher Scientific), 100 U/ml penicillin, 100 μg/ml streptomycin, and 1 mM CaCl_2_ (Merck KGaA, Darmstadt, Germany) and used for further culturing for 6 weeks.

### Proliferation assays

Cell proliferation assays were ATP-based and performed using the BioFix® Lumi ATP assay (Macherey-Nagel, Düren, Germany) according to the manufacturer’s protocol.

### RNA isolation and cDNA synthesis

Total RNA of hiPSC and spontaneously beating clusters of differentiated hiPSC cultured for 10–12 days (dhiPSC) and 8 weeks (pacemaker cell cluster (PCC)), respectively, were isolated using Trizol® reagent (Thermo Fisher Scientific) following the manufacturer’s instructions. RNA of the human right atrium (hRA) was purchased (BioCat GmbH). cDNA was synthesized by reverse transcription according to the manufacturer’s protocol (Superscript II; Thermo Fisher Scientific).

### Quantitative real-time polymerase chain reaction (qRT-PCR)

qRT-PCR was performed using an ABS 7500 Realtime PCR System (Thermo Fisher Scientific) according to the manufacturer’s protocol. Ninety-six-well optical detection plates (Thermo Fisher Scientific) were loaded to a total volume of 10 μl per well, consisting of 0.5 μl cDNA, 5 μl TaqMan Fast Universal Master Mix (Thermo Fisher Scientific), and 6-carboxyfluorescein (FAM)-labeled pre-designed TaqMan probes and primers (TaqMan Gene Expression Assays, Thermo Fisher Scientific) (Additional file 3). In addition, pre-designed primers and probes detecting the housekeeping genes glyceraldehyde 3-phosphate dehydrogenase (GAPDH), hypoxanthine-guanine phosphoribosyltransferase 1 (HPRT1), and beta-actin (ACTB) were applied and normalization was carried out by a modified threshold cycle (CT) relative quantification method using the three housekeeping genes, as published elsewhere [21]. All PCR reactions were performed in triplicate and data were expressed as an average of triplicates. To compare relative abundance of gene expression in hiPSC, dhiPSC, and PCC with human cardiac tissue, we analyzed relative abundance of gene transcripts in the hRA (BioCat). Based on previously published levels of hRA and SAN transcripts for selected genes [22], human SAN (hSAN) transcript levels were estimated in order to evaluate the differentiation status of hiPSC.

### Immunocytochemistry

#### Preparation of hiPSC

MEF were seeded on coverslips of 1.2-cm diameter (Menzel-Gläser, Gerhard Menzel GmbH, Braunschweig, Germany). Coverslips were previously transferred to a 24-well plate and coated with 100 μg/ml fibronectin (Roche Applied Science, Penzberg, Germany)/phosphate-buffered saline (PBS; Thermo Fisher Scientific) solution for 30 min at 37 °C. The next day, hiPSC colonies were added and cultured in hiPSC medium (for details please refer to Additional file 1). When they had grown adherent, hiPSC were rinsed in PBS, fixed in 1% formaldehyde (Merck)/PBS for 15 min at room temperature and stored in 0.1% formaldehyde/PBS at 4 °C.

#### Preparation of PCC

Eight-week-old PCC were picked, embedded in Tissue-Tek™ (Thermo Fisher Scientific), frozen, and sliced into 5-μm sections at –15 °C to –20 °C using a cryotome (Microtome 5030; Bright Instrument Co Ltd, Huntingdon, UK) followed by storage at –20 °C.

#### Preparation of dispersed pacemaker cells

PCC were enzymatically and mechanically dissociated at week 8. In detail, PCC were washed in PBS, detached from the dish surface using a scalpel and incubated in 1 mg/ml collagenase type IA (Sigma-Aldrich)/PBS for 30 min at 37 °C. After incubation, each PCC was transferred on a fibronectin-coated 0.5-cm diameter coverslip (Menzel-Gläser) and covered with Medium 199. Using a 10-μl sterile micropipette tip (Eppendorf AG, Hamburg, Germany), PCC were gently dissociated. Individual cells were allowed to attach on the coverslip overnight before immunocytochemistry assays were performed.

For immunocytochemistry, probes were incubated with 0.5% TritonTM X-100 (Sigma-Aldrich)/PBS solution for 15 min, treated with 0.1 M glycine (Merck)/PBS solution for 60 min, and incubated in blocking solution (2% bovine serum albumin; Sigma-Aldrich) in PBS for 2–3 h. Primary antibodies (Additional file 4) were diluted 1:200 in blocking solution and incubated at 4 °C overnight. The next day, sections were rinsed with PBS (3 × 2 min) and incubated with secondary antibodies (Additional file 4) diluted 1:200 in blocking solution at 4 °C for 4 h. Cells were mounted with Citifluor glycerol/PBS solution AF1 (Agar Scientific Ltd., Stansted, UK) and analyzed by fluorescence microscopy using an Olympus IX 70 fluorescence microscope with a peltier-cooled CCD camera (Olympus, Tokyo, Japan) and confocal microscopy using a Leica TCS SP8 laser-scanning confocal microscope (Leica Microsystems, Wetzlar, Germany) connected to an inverted microscope (DMI-6000) equipped with a 63× oil immersion lens (NA 1.4). Images were processed using Adobe Photoshop (Adobe Systems Incorporated, San José, CA, USA) and ImageJ software (National Institutes of Health, Bethesda, MD, USA) using standard quantification methods.

### Multi-electrode array recordings and pharmacological stimulation/inhibition

To record firing rate and analyze autonomic response a multi-electrode array (MEA) system (Multichannel Systems, Reutlingen, Germany) was used. PCC were plated on laminin/fibronectin-coated MEAs (60MEA200/30iR-Ti, Multichannel Systems) and cultured for 3 days to achieve attachment to the electrodes. Electrical signals (extracellular field potentials) were recorded in Medium 199 (Sigma-Aldrich) supplemented with 1 mM CaCl_2_ media at 37 °C and sampled at 20 kHz with the MEA_Rack software (Multichannel Systems). Signals were analyzed using Spike2 software (v7, Cambridge Electronic Design, Cambridge, UK). PCC underwent β-adrenergic (1 μM isoproterenol; Sigma-Aldrich) and muscarinic (1 μM carbachol; Sigma-Aldrich) challenge to study the autonomic capacity to modulate firing rate (*n* = 6, each). Moreover, different concentrations of ivabradine (1, 3, 50 μM; Sigma-Aldrich) were applied to assess effects of I_f_ blockage (*n* = 6, each).

### Co-cultures of PCC and neonatal rat ventricular myocytes

Ventricular myocytes from 1-day-old neonatal rats (Crl:Wls River, strain code 003, coat color white, Charles River Laboratories, Wilmington, MA, USA) were prepared as previously reported [23]. Neonatal rat ventricular myocytes (NRVM) were cultured in Medium 199 supplemented with 10% FBS, 100 U/ml penicillin, 100 μg/ml streptomycin, and 1 mM CaCl_2_. Eight-week-old PCC were seeded on 35-mm dishes (ibidi, Martinsried, Germany), with each dish containing a single PCC. On the following day, 10^6^ NRVM per dish were added to half of the dishes for co-culture experiments, while the other dishes comprised single PCC (*n* = 10, each). Electrical integration of the PCC within the monolayer was evaluated based on synchronous contraction of the whole culture. For control, NRVM monocultures at the same concentration (10^6^ per dish, *n* = 10) were recorded in parallel. Co-cultures of PCC and NRVM as well as NRVM monolayers (*n* = 10, each) were stimulated with 1 μM isoproterenol (Sigma-Aldrich) 1 week after culture onset, respectively, to assess responsiveness to adrenergic stimulation and capacity to modulate the rate. For evaluation of beating rate, dishes were kept under 37 °C conditions and computerized beat rate measurements were performed 15 s per microscope section every second day or after pharmacological application using a Fourier-analysis (program software “Imagoquant”, Mediquant, Halle/Saale, Germany).

### Recording of calcium transients

Pacemaker cell clusters (PCC) were loaded with the Ca^2+^-sensitive fluorescent indicator fluo-4-AM for 10 min at 37 °C. After 10 min of de-esterification, line-scan images were taken in spontaneously beating cells of clusters on a confocal microscope (Olympus FluoView1000). Fluo-4 was excited at 473 nm and emission was collected above 500 nm. Temporal resolution was 10 μs per pixel. Line-scan images were processed using ImageJ (National Institutes of Health) and data were analyzed in OriginPro (OriginLab, Northampton, MA, USA).

### Recording of spontaneous action potentials from individual cells within PCC

PCC were dissociated to isolate individual cells at week 8 as previously described (please refer to the previous paragraph on immunocytochemistry). For patch-clamp recordings, glass coverslips with a diameter of 5 mm were coated with 0.1% gelatin and placed into each well of a 96-well plate. Spontaneously beating, individual cells were used. Data were recorded at 37 °C using the whole-cell patch-clamp technique and action potential (AP) recordings were obtained in the current clamp configuration [24]. Bath solution was composed of Medium 199 supplemented with 10% FBS, 100 U/ml penicillin, 100 μg/ml streptomycin, and 1 mM CaCl_2_. Pipette solution consisted of 145 mM KCl (Merck), 5 mM NaCl (Merck), 2 mM CaCl_2_, 4 mM EGTA (Merck), 2 mM MgCl_2_ (Merck), and 10 mM HEPES (Sigma-Aldrich). Pipettes had resistances between 1.5 and 5 MΩ. Data were low-pass filtered at 1 to 2 kHz (–3 dB, four-pole Bessel filter) before digitalization at 5 to 10 kHz. Recordings were performed using a commercially available amplifier (Warner OC-725A, Harvard Apparatus, Holliston, MA, USA) and pCLAMP software (Molecular Devices, LLC, Sunnyvale, CA, USA) for data acquisition and analysis.

### Statistical analysis

All experiments and primary analyses were blinded. Statistical analysis was performed using GraphPad Prism (Version 6.0, Graphpad Software, Inc., San Diego, CA, USA). Comparison between multiple groups was performed using one-way analysis of variance (ANOVA) followed by a Tukey post-hoc test. Pairwise comparison was performed by two-tailed unpaired Student’s *t* test. Differences were considered significant at the level *p* < 0.05. Data are presented as arithmetic mean ± standard error of the mean (SEM).

## Results

### Differentiation of hiPSC colonies into PCC

**Fig. 1:**
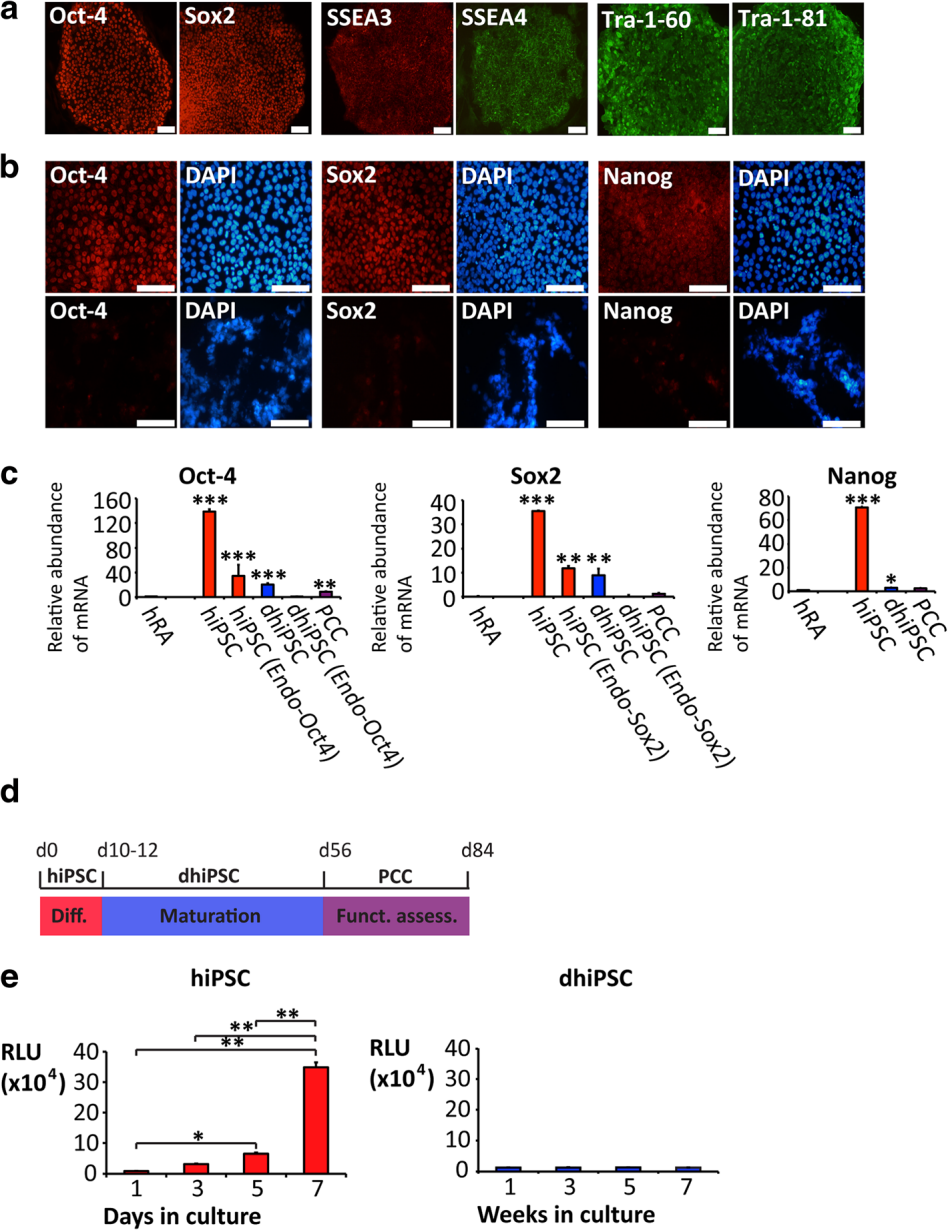
Differentiation of hiPSC colonies to pacemaker cell clusters. **a** Immunostaining of undifferentiated hiPSC shows typical morphology and provides evidence of pluripotency markers. **b** Positive immunostaining of undifferentiated hiPSC colonies for pluripotency markers (*top*) and absence of staining in clusters differentiated by co-culture (dhiPSC) (*bottom*). **c** Transcription of pluripotency markers in hRA, undifferentiated hiPSC, dhiPSC, and PCC. **d** Differentiation/maturation protocol: hiPSC and END-2 cells were co-cultured for 10–12 days (dhiPSC) followed by transfer to an FBS-enriched culture medium for further differentiation/maturation (PCC; altogether 56 days). Subsequently, functional analysis was undertaken in a co-culture model with neonatal rat ventricular myocytes (NRVM) (28 days). **e** Proliferation assay of undifferentiated hiPSC (*top*) and throughout the differentiation/maturation process (*bottom*). *Scale bars* = 100 μm. Data are provided as means ± SEM. *p < 0.05, **p < 0.01, ***p < 0.001, versus hRA; comparison between multiple groups was performed using one-way ANOVA followed by a Tukey post-hoc test. Statistical significance compared to hiPSC is not indicated (*p* < 0.01 in each panel). *hRA* human right atrium (dark green), *hiPSC* human induced pluripotent stem cells (red), *dhiPSC* co-culture differentiated hiPSC (blue), *PCC* pacemaker cell clusters (purple), *RLU* relative light units


Differentiation experiments were performed with three different hiPSC lineages originating from human fibroblast cultures that were obtained from skin biopsies of healthy control probands. Data originating from line #1 are depicted in the main paper; replications using lines #2 and #3 are described in Additional file 5. hiPSC colonies were propagated in the undifferentiated state on top of a MEF feeder layer. Cells grew with typical morphology, expressed the pluripotency markers Oct-4, Sox2, Nanog, Tra-1-60, Tra-1-81, SSEA3, and SSEA4 (Fig. 1a and b) and were able to form teratomas after subcutaneous injection into immunodeficient mice (Additional file 2). For cardiomyogenic differentiation, hiPSC were co-cultured with END-2 cells [6] under serum-free culture conditions [25]. Spontaneously beating clusters could be observed from co-culture day 10 onwards. At day 12, approximately 60% of the initial hiPSC colonies showed regular contractions. Continued co-culture differentiation resulted in the formation of cardiomyogenic cell clusters that yielded predominantly ventricular-type AP properties and were characterized by spontaneous rates of ~ 40 beats/min [6] (Additional file 6). In an effort to avoid working-type myogenic differentiation, early hiPSC-derived clusters (dhiPSC) were dissected immediately after beating onset (days 10–12) and transferred to dishes containing a FBS (10%)-enriched medium for continuous culturing for 8 weeks, followed by a further culturing period for functional characterization (4 weeks). Immunostaining and qRT-PCR demonstrated that pluripotency markers were downregulated upon co-culture differentiation for 10–12 days (Fig. 1b and c) and remained low throughout further culturing in FBS-enriched medium (Fig. 1c). The firing rate of clusters continuously increased with beating onset (Additional file 6) and stabilized at a total of 8 weeks in culture, characterized by robust and regular rates (70–90 beats/min) (Additional file 5; please also refer to the movie files in Additional files 7 and 8). Clusters were thus designated as pacemaker cell clusters (PCC) (Fig. 1d) and were subjected to in-depth cellular investigation. First, we performed adenosine triphosphate (ATP)-based proliferation assays of native hiPSC followed by assays throughout the differentiation and maturation process. While proliferative activity was high in hiPSC, differentiated cell lines showed negligible proliferation (Fig. 1e).

Additional file 7: Movie of spontaneously beating PCC derived from hiPSC line #1.


### Culture-based differentiation induces activation of a pacemaker-related gene program

We aimed to elucidate the transcription profiles underlying spontaneous activity of cells treated by co-culture differentiation for 10–12 days (dhiPSC) and after further culturing in the presence of FBS up to week 8 (PCC). Transcript levels were compared to native, non-beating hiPSC. Gene transcription in a pool of commercially available human right atrial samples served as a reference. As hSAN tissue was not available, hSAN transcript levels were calculated based on previously published data [22] and utilized to evaluate the differentiation status of cell clusters.

#### Pacemaker-specific transcription factors

**Fig. 2:**
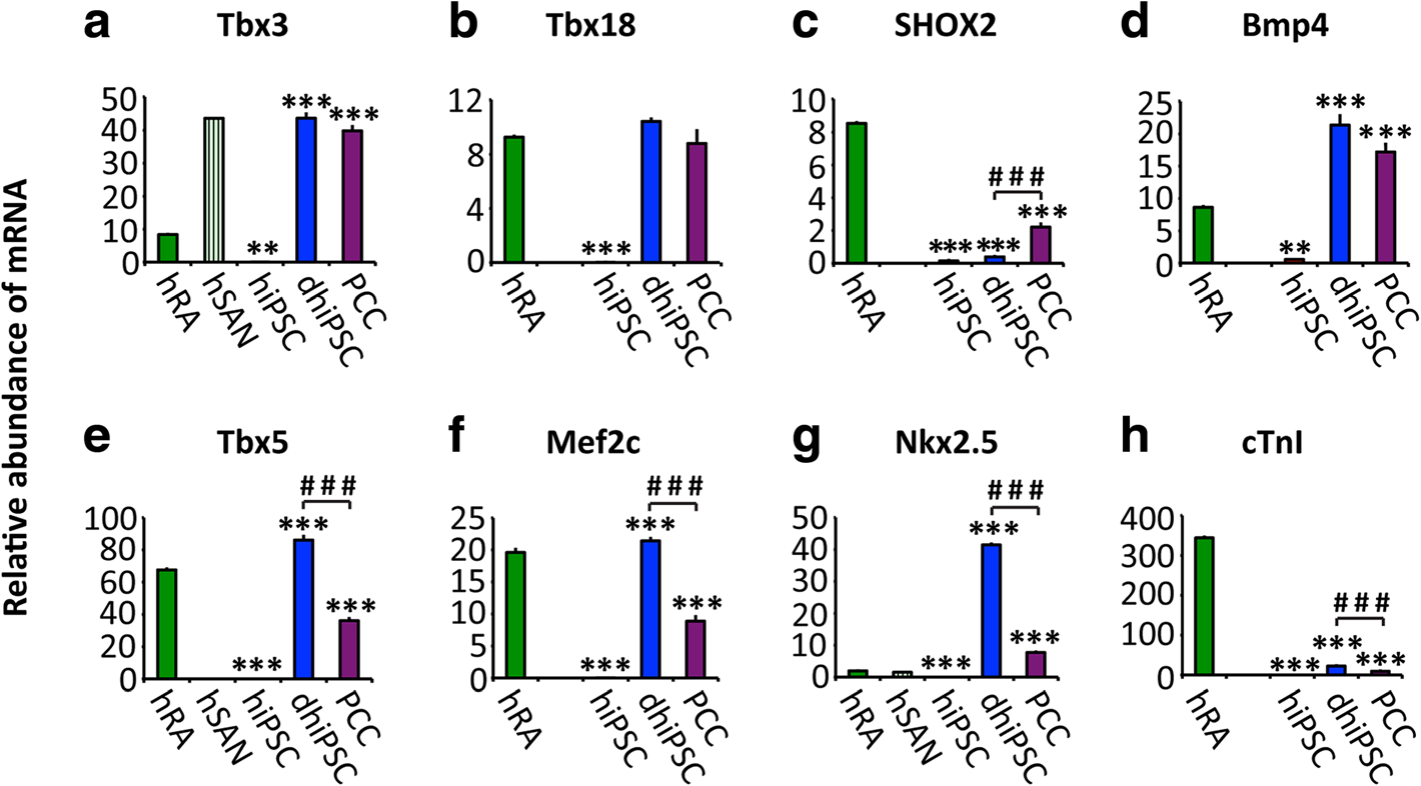
Transcription profiling of pacemaker- and working-type specific regulators and markers. Relative abundance of mRNA transcription, analysed by qRT-PCR. **a**–**d** SAN-related transcription factors; **e**–**g** myocardium-related transcription factors; **h** myocardial marker troponin I (*cTnI*). Data are provided as means ± SEM. *p < 0.05, **p < 0.01, ***p < 0.001, versus hRA; ^#^ *p* < 0.05, ^##^ *p* < 0.01, ^###^ *p* < 0.001, dhiPSC versus PCC; comparison between multiple groups was performed using one-way ANOVA followed by a Tukey post-hoc test. Statistical significance compared to hiPSC is not indicated (*p* < 0.01 in every panel). *hRA* human right atrium (dark green), *hSAN* human sinoatrial node (green shaded), *hiPSC* human induced pluripotent stem cells (red), *dhiPSC* co-culture differentiated hiPSC (blue), *PCC* pacemaker cell clusters (purple)

T-box transcription factors 3 (Tbx3) and 18 (Tbx18) contribute importantly to the development of pacemaker sites by suppression of ventricular cardiomyocyte differentiation, activation of nodal-specific genetic pathways [1], and initiation of SAN formation [26], respectively. Transcripts of both Tbx3 and Tbx18 were virtually absent in hiPSC (Fig. 2a and b) but increased significantly upon differentiation (1146-fold for Tbx3, *p* < 0.001; 582-fold for Tbx18, *p* < 0.001) and remained high throughout further culturing in FBS-enriched medium. Compared to transcription in the hRA, levels were markedly higher (five-fold for Tbx3, *p* < 0.001) or similar (for Tbx18, *p* = 0.36; Fig. 2a and b). Furthermore, we analyzed the transcription factor SHOX2 and its target Bmp4, involved in pacemaker cell development [27]. SHOX2 was significantly upregulated by culturing in the FBS-enriched medium (15-fold, *p* < 0.001), although absolute gene expression remained lower than in the atrium (four-fold, *p* < 0.001; Fig. 2c). Bmp4 transcripts were barely detectable in hiPSC, but increased markedly leading to levels in early dhiPSC clusters and in PCC that exceeded the levels in the human right atrium (two-fold, *p* < 0.001, for both; Fig. 2d).

#### Myocardial transcription factors and marker genes

Transcription factors Tbx5, Nkx2.5, and Mef2c are involved in differentiation and structural maturation of ventricular cardiomyocytes [28]. While Tbx5 and Nkx2.5 both promote ventricular development [29], overexpression of Nkx2.5 represses SAN development [30], indicating a reverse role in nodal-type cell differentiation. In native hiPSC colonies, transcripts of Tbx5, Nkx2.5, and Mef2c were not detected but abundant transcription was observed after co-culture differentiation (2255-fold increase for Tbx5, *p* < 0.001; 4580-fold increase for Nkx2.5, *p* < 0.001; 958-fold increase for Mef2c, *p* < 0.001; Fig. 2e–g). Gene transcription of these working-type myocardial markers was significantly downregulated after further culturing in FBS-enriched medium (two-fold decrease for Tbx5, *p* < 0.001; five-fold decrease for Nkx2.5, *p* < 0.001; two-fold decrease for Mef2c, *p* < 0.001; Fig. 2e–g). In line with this, cardiac troponin I (cTnI) levels were absent from hiPSC and were low after co-culture and FBS-enriched culturing (Fig. 2h), indicating a lack of working-type myocardial differentiation.

#### Ion channels and transporters

**Fig. 3:**
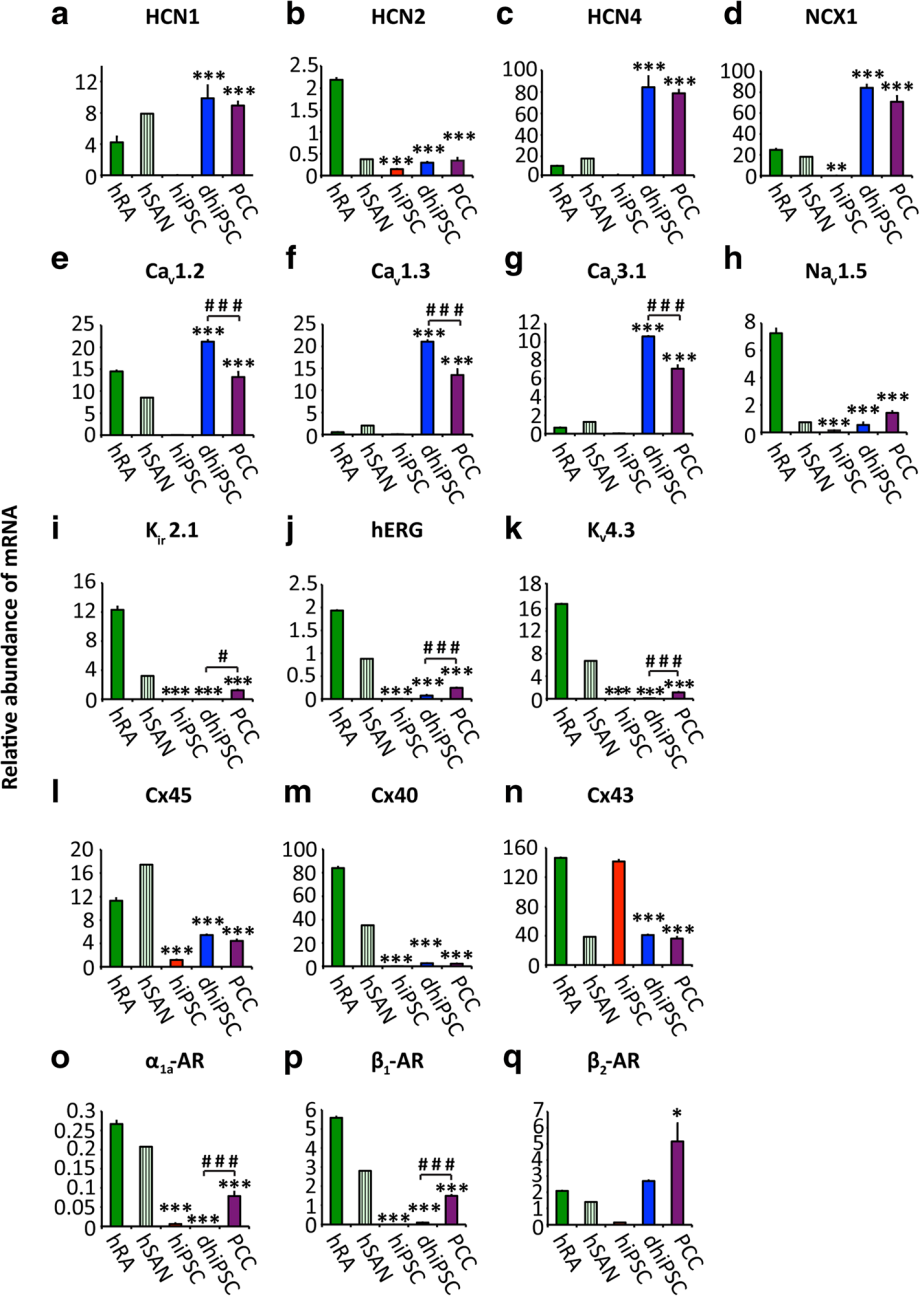
Transcription profiling of ion channels, transporters, connexins, and adrenergic receptors. Relative abundance of mRNA transcription analyzed by qRT-PCR. **a**–**c** Hyperpolarization-activated cyclic nucleotide channels (*HCN*); **d** Na-Ca exchanger 1 (*NCX1*); **e**–**g** calcium channels; **h** sodium channel Na_v_1.5; **i**–**k** potassium channels; **l**–**n** connexins (*Cx*); **o**–**q** adrenergic receptors (*AR*). Data are provided as means ± SEM. *p < 0.05, **p < 0.01, ***p < 0.001, versus hRA; ^#^ *p* < 0.05, ^##^ *p* < 0.01, ^###^ *p* < 0.001, dhiPSC versus PCC; comparison between multiple groups was performed using one-way ANOVA followed by a Tukey post-hoc test. Statistical significance compared to hiPSC is not indicated (*p* < 0.01 in every panel). *hRA* human right atrium (dark green), *hSAN* human sinoatrial node (green shaded), *hiPSC* human induced pluripotent stem cells (red), *dhiPSC* co-culture differentiated hiPSC (blue), *PCC* pacemaker cell clusters (purple)

Hyperpolarization-activated cyclic nucleotide channels (HCN) (Fig. 3a–c) produce the pacemaker current (I_f_) that is importantly involved in SAN pacemaker activity [31, 32] and early embryonic heart development [17, 33]. Hardly detectable in hiPSC, transcripts of nodal-specific HCN isoforms 1 and 4 [34] were significantly upregulated upon hiPSC co-culture differentiation (329-fold for HCN1, *p* < 0.001; 129-fold for HCN4, *p* < 0.001), reaching levels similar or higher compared to the hSAN. High levels of transcription were maintained in PCC (*p* = 0.89 for HCN1; *p* = 0.87 for HCN4; Fig. 3a and c). The sodium calcium exchanger 1 (NCX1), crucial for SAN function [35], was significantly upregulated upon co-culture differentiation (1981-fold, *p* < 0.001) and reached transcription levels in PCC that even exceeded hSAN levels (Fig. 3d). Transcript levels of l-type calcium channel subunits Ca_v_1.2 and Ca_v_1.3 and the T-type subunit Ca_v_3.1 were almost absent from hiPSC, but increased markedly throughout co-culture differentiation (540-fold for Ca_v_1.2, *p* < 0.001; 368-fold for Ca_v_1.3, *p* < 0.001; 369-fold for Ca_v_3.1, *p* < 0.001) and showed levels similar or higher than in the atrium/hSAN after culturing in FBS-enriched medium (Fig. 3e–g). Na_v_1.5, underlying the main depolarizing cardiac sodium current in working-type myocytes [36], showed transcript levels in PCC significantly lower than in the atrium (five-fold, *p* < 0.001; Fig. 3h). Transcripts of K channel genes encoding the inward rectifier subunit K_ir_2.1, hERG, and the K_v_4.3 subunit were not detected in hiPSC and remained low upon differentiation (Fig. 3i–k).

#### Connexins (Cx)

Spatial connexin expression contributes essentially to the electrophysiological properties of specified cardiac structures [37]. While Cx45 is characteristic for the SAN and the conduction system [38], Cx40 and Cx43 subunits represent components of the working myocardium [37, 38]. hiPSC displayed high transcript levels of Cx43, and low levels of Cx40 and Cx45 (Fig. 3l–n). Further differentiation in FBS-enriched medium resulted in marked downregulation of Cx43 (four-fold, *p* < 0.001), while Cx40 levels remained low (*p* = 0.11) and Cx45 levels were significantly increased (four-fold, *p* < 0.001) (Fig. 3l–n).

#### Adrenergic receptors

Adrenergic receptors are critical for modulation of rate response in the SAN [39]. Relevant transcripts of adrenergic receptors were not detected in hiPSC (Fig. 3o–q). Upon differentiation, α_1a_- and β_1_-adrenoceptor transcripts remained low during co-culturing, but were markedly increased throughout further culturing in FBS-enriched medium (12-fold for α_1a_-adrenoceptor, *p* < 0.001; 295-fold for β_1_-adrenoceptor, *p* < 0.001; Fig. 3o and p). β_2_-adrenoceptors were upregulated during co-culture, reaching levels similar to the human right atrium (*p* = 0.87) or hSAN, while expression further increased under the influence of FBS up to week 8 (two-fold, *p* = 0.02) (Fig. 3q).

#### Membrane expression of pacemaker-related ion channels and connexins

**Fig. 4:**
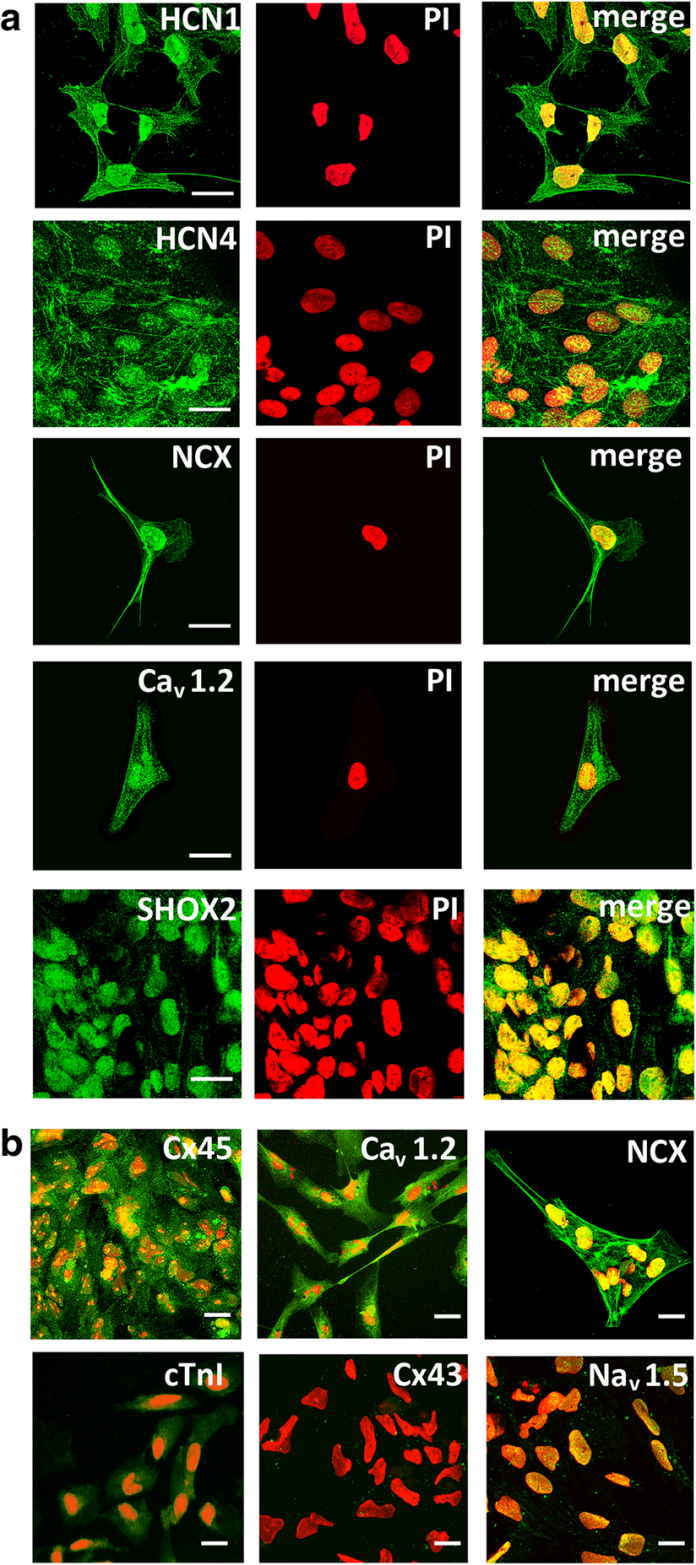
Immunocytochemical analysis of cells dispersed from pacemaker cell clusters (PCC). Signals were visualized by confocal (**a**) and fluorescence microscopy (**b**). Cells were immunolabeled and imaged by using identical protocols. **a** Left column (from top to bottom): anti-HCN1, anti-HCN4, anti-NCX, and anti-Ca_v_1.2 staining indicates distinct membrane expression of the respective proteins. Note that anti-SHOX2 immunolabeling is concentrated to the nucleus. Middle column: nuclei of respective samples are counterstained with propidium iodide (*PI*). Right column: overlay of immunostains and PI counterstain. **b** Fluorescence microscopic visualization of anti-Cx45, anti-NCX, and anti-Ca_v_1.2 in less dispersed areas of PCC, showing abundant membrane expression. In contrast, anti-cTnI immunolabeling reveals only little signal intensity, while images of other working-type cardiomyocyte marker proteins display no specific membrane signal (Cx43, Nav1.5). Nuclei are counterstained with PI (*red*). *Scale bars* = 20 μm. *cTnI* cardiac troponin I, *Cx* connexin, *HCN* hyperpolarization-activated cyclic nucleotide channels, *NCX* sodium calcium exchanger

While hiPSC colonies are characterized by minor membrane expression of pacemaker-related ion channels and connexins, immunocytochemical analysis of cells dispersed from PCC revealed marked membrane signals of HCN1, HCN4, NCX, Ca_v_1.2, and Cx45 (Fig. 4). Moreover, extensive nuclear signal of the nodal-specific transcription factor SHOX2 could be observed, further substantiating the nodal phenotype. In contrast, low signals for cTnI and negligible membrane expression of Cx43 and Nav1.5 (Fig. 4b), which represent typical marker proteins of working-type cardiomyocytes, confirmed the specificity of pacemaker-type cells. This is underlined by immunohistochemical analysis of PCC using “whole mount” cryosections (Additional file 9).

### Functional and pharmacological characteristics of PCC

**Fig. 5:**
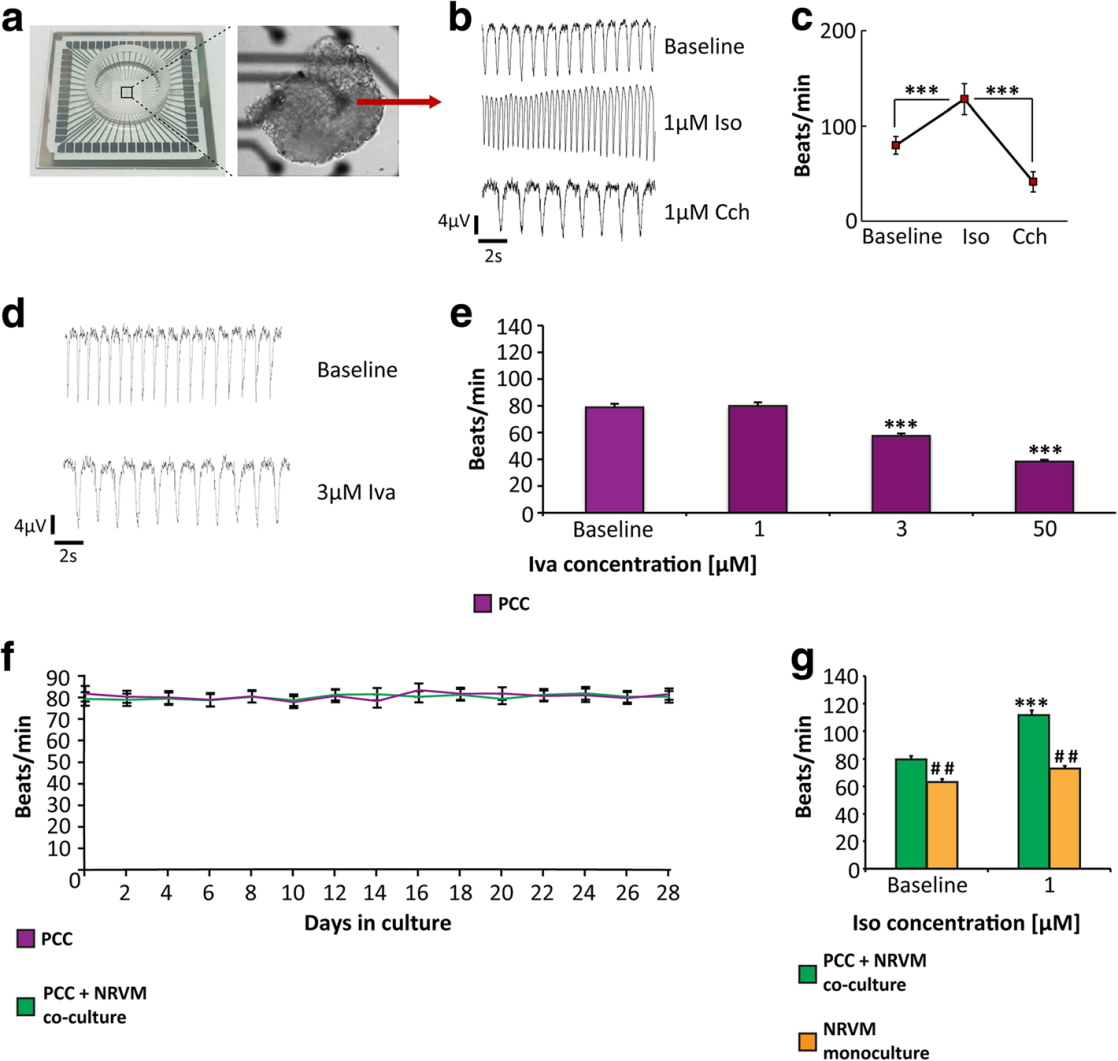
Rate profiles and pharmacological stimulation of pacemaker cell clusters (*PCC*). **a** Layout of the experimental design for PCC functional characterization using a multi-electrode array (MEA) system. **b** Representative raw traces from an electrode of a MEA plated with a spontaneously beating PCC showing field potentials at baseline and after change to medium containing the β-adrenergic agonist isoproterenol (*ISO*, 1 μM). Subsequent muscarinic challenge using carbachol (*Cch*, 1 μM) significantly slowed the firing rates. **c** Average firing rates recorded from MEAs demonstrate chronotropic response of PCC to stimulation with autonomic substances (*n* = 6 per group). **d** Representative traces of PCC field potentials at baseline and after application of the I_f_ inhibitor ivabradine (*IVA*, 3 μM). **e** Dose-dependent rate reduction of PCC upon stimulation with IVA (*n* = 6 per group). **f** Rate profiles of spontaneously beating PCC (*n* = 10, purple) compared to co-cultures of PCC and neonatal rat ventricular myocytes (*NRVM*) (*n* = 10, blue) over an observation period of 28 days. **g** Rate response upon ISO stimulation. Co-cultures (*n* = 10, green) of PCC and NRVM exhibited higher rates and chronotropic response upon ISO stimulation than NRVM monolayers (*n* = 10, orange) at culture day 4. Data are provided as means ± SEM. *p < 0.05, **p < 0.01, ***p < 0.001, pharmacologically stimulated samples versus baseline control; ^#^ *p* < 0.05, ^##^ *p* < 0.01, ^###^ *p* < 0.001, PCC samples versus PCC + NRVM co-cultures; comparison between multiple groups was performed using one-way ANOVA followed by a Tukey post-hoc test

hiPSC-derived pacemaker cell clusters (PCC) cultured over a period of 8 weeks according to our protocol (Fig. 1d) exhibited regular contractions and constant rates (Fig. 5a–f; see Additional files 7 and 8 for movies), which remained stable throughout a subsequent observational period of 28 days (Additional file 5). SAN pacemaker cells change firing rates according to autonomic input. To assess adrenergic and cholinergic rate response, PCC were plated on MEAs to record extracellular field potentials (Fig. 5a). Beta-adrenergic stimulation (1 μM isoproterenol) increased the firing rates of PCC from 78.9 ± 4.2 beats/min to 129.8 ± 8.9 beats/min (*n* = 6; *p* < 0.001; Fig. 5b and c). Subsequent exposure to carbachol (1 μM) decreased the rate to 42.2 ± 5.1 beats/min (*n* = 6; *p* < 0.001; Fig. 5b and c). Treatment of PCC with ivabradine at a dosage that specifically inhibits the f-current [40] elicited significant decline in the beating rate (∆ rate of –22.5 ± 2.3 beats/min using 3 μM ivabradine; *n* = 6; *p* < 0.001; Fig. 5d and e).

### PCC pace neonatal rat ventricular myocytes in co-culture experiments

When co-cultured with neonatal rat ventricular myocytes (NRVM), PCC initiated a constant and synchronized beating of co-cultures (79.9 ± 2.8 beats/min; *n* = 10) similar to that of monocultured PCC (Fig. 5f) that was maintained during follow-up for 28 days. Notably, neonatal myocyte monolayers showed significantly slower beating rates (66.7 ± 2.1 beats/min; *n* = 10; *p* < 0.01; Fig. 5g), which declined after culturing for 2 weeks to rates < 40 beats/min. Furthermore, rate response after stimulation with isoproterenol (1 μM) was significantly higher in co-culture of PCC with neonatal myocytes compared to neonatal myocyte monolayers (Fig. 5g).

### PCC elicit spontaneous Ca^2+^ transients

**Fig. 6:**
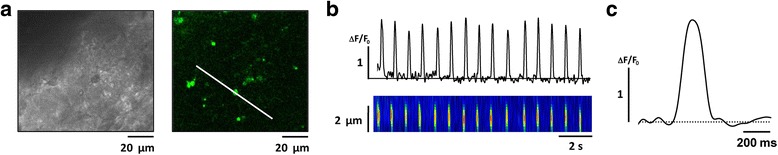
Spontaneous Ca^2+^ transients recorded in beating PCC. **a** Transmission light and confocal images of fluo-4-AM loaded PCC. Line-scan images (indicated by *white line*) were taken at 10 μs per pixel; line-scan and line profile of a representative recording are depicted in (**b**). Note the regularity of the spontaneous transients. **c** Enlargement of a single Ca^2+^ transient of the trace shown in (**b**)

While membrane channels importantly contribute to pacemaking, SAN cells are also triggered by the finely orchestrated interplay of ion channels with intracellular Ca^2+^ cycling leading to spontaneous Ca^2+^ transients. Confocal line-scan images of changes in [Ca2+]_i_ in fluo-4-AM loaded PCC demonstrated regular repetitive oscillations in [Ca^2+^]_i_ indicating spontaneous Ca^2+^ transients (Fig. 6). This suggests a “coupled clock” mechanism that drives automaticity in pacemaker-type cells.

### Morphological features and action potential properties of cells isolated from PCC

**Fig. 7:**
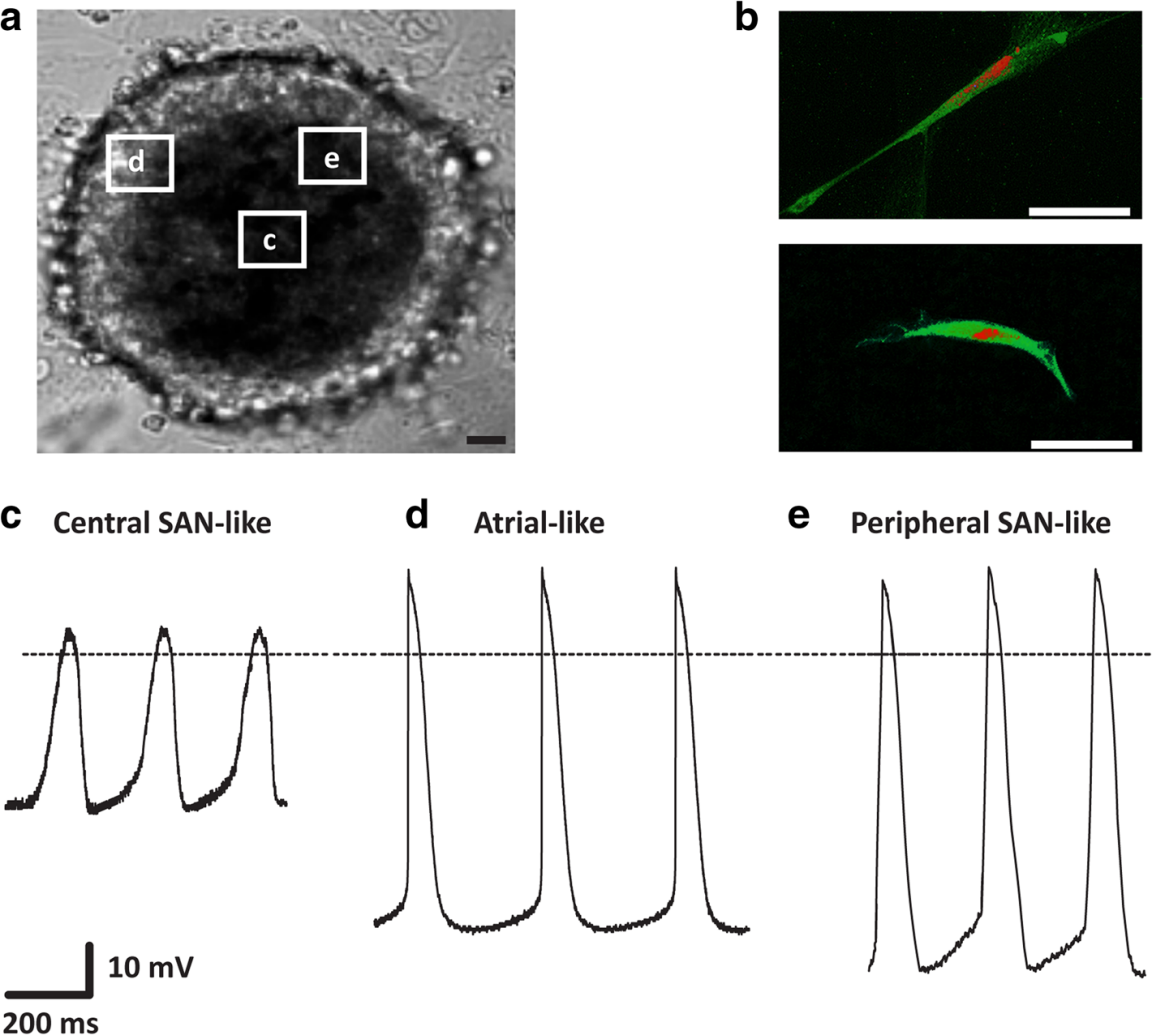
Morphological and electrophysiological characteristics of cells isolated from PCC. **a** Typical aspect of a spontaneously beating PCC. Origin of isolated cells characterized by patch-clamp recordings are framed for illustration. *Scale bar* = 50 μm. **b** Single cells isolated from PCC center, immunostained with anti-HCN4 (*green*) and counterstained with propidium iodide (*red*) showed typical elongated cell morphology and intense membrane expression of HCN4. Signals were visualized by confocal microscopy. *Scale bars* = 50 μm. **c**–**e** Spontaneous action potentials (APs) (*n* = 41) were recorded from individual cells dissociated from PCC using the whole-cell voltage-clamp technique. Representative recordings are depicted and show AP morphologies resembling (**c**) central sinoatrial node (*SAN*) (*n* = 21), (**d**) atrial (*n* = 15), and (**e**) peripheral SAN (*n* = 5) cells

We next assessed morphological and electrophysiological features of cells isolated from PCC. Native SAN pacemaker cells have a specific appearance, characterized by an elongated, spindle-shaped morphology and pronounced membrane expression of HCN4, which serves as marker protein of the SAN [41]. Cells isolated from the center of PCC (Fig. 7a) resembled native SAN cells with respect to their elongated shape and abundant membrane expression of HCN4 (Fig. 7b). To analyze electrophysiological properties, spontaneous APs were recorded from individual cells dissociated from PCC (Fig. 7a). We sought to characterize cell subtypes by determining rate interval, maximum diastolic potential (MDP), peak voltage, amplitude (Amp), maximal rate of depolarization (dV/dt_max_), AP duration (APD) at different levels of repolarization (APD measured at 10% increments of Amp), and APD_30–40_/APD_70–80_ ratio (with ratios > 1.5 indicating ventricular-type APs) [42] (Table 1). Based on these criteria, 21 of 41 cells displayed APs resembling typical nodal-type characteristics of central SAN myocytes (Fig. 7c). Parameters comprised short beat rate interval, low AP amplitudes, a maximal diastolic potential that was less negative than in working-type cardiomyocytes, and an APD_30–40_/APD_70–80_ ratio of 1.0 ± 0.1, consistent with AP morphology without a plateau phase (Fig. 7c, Table 1). Fifteen of 41 cells showed AP properties attributed by longer beating rate interval and AP amplitude, a maximal diastolic potential that was more negative than in central SAN myocytes, and a shorter AP duration with faster maximal rate of depolarization typical for atrial-type APs (Fig. 7d, Table 1). Furthermore, 5 of 41 cells showed AP configurations between central SAN- and atrial-type previously designated as peripheral SAN-type APs (Fig. 7e, Table 1) [43]. Interestingly, pacemaker-type cells within the PCC appeared structurally organized. Central SAN-type AP morphology was primarily shown by cells localized at the center of a cluster, while peripheral cells were characterized by atrial- or peripheral SAN-type APs (Fig. 7a–e).

**Table 1:** Electrophysiological characterization of action potentials recorded from isolated cells.

Action potentials (*n* = 41)	Interval (ms)	MDP (mV)	Peak (mV)	Amp (mV)	dV/dt_max_ (mV)	APD_30_ (ms)	APD_40_ (ms)	APD_70_ (ms)	APD_80_ (ms)	APD_30-40/_ APD_70-80_
Central SAN-like (*n* = 21)	400.0 ± 6.9	–40.4 ± 4.2	7.3 ± 0.9	47.8 ± 3.5	1.0 ± 0.1	139.0 ± 9.7	162.3 ± 11.1	173.7 ± 11.9	196.3 ± 13.4	1.0 ± 0.1
Atrial-like (*n* = 15)	476.6 ± 22.3	–77.2 ± 1.2	22.2 ± 1.3	99.4 ± 0.7	27.7 ± 1.1	77.4 ± 1.7	89.3 ± 2.0	107.2 ± 2.4	131.0 ± 2.9	1.1 ± 0.2
Peripheral SAN-like (*n* = 5)	406.4 ± 7.9	–79.4 ± 22.2	27.5 ± 3.0	106.9 ± 2.2	4.9 ± 0.4	71.3 ± 3.5	83.7 ± 3.9	100.5 ± 4.7	112.1 ± 2.9	1.1 ± 0.1

Data are provided as means ± SEM

*Amp* amplitude, *APD* AP duration, *dV/dt*_*max*_ maximal rate of depolarization, *Interval* beating rate interval, *MDP* maximum diastolic potential, *Peak* peak voltage, *SAN* sinoatrial node

## Discussion

The generation of biological pacemaker activity may offer a promising avenue to overcome the limitations of electronic pacemaker devices. To date, scientific approaches essentially comprise two strategies: 1) the use of cell replacement therapy to substitute loss of active pacemaker cells; and/or 2) viral transfection with genes that either transform myocytes into virtual pacemaker cells or elicit currents that produce spontaneous excitation of previously quiescent cells [10, 14, 15, 41, 44–46]. Concerns remain with respect to hazards such as immune rejection, tumor genesis, arrhythmogenicity, or only transient effects of pacemaker function [14, 15, 47]. Moreover, insufficient beating rates and lack of complete differentiation/integration of generated, electrically active cells into pacemaker sites constitute challenges that need to be overcome when seeking clinical translation. The ability of hiPSC to differentiate into various types of cardiac cells that could be retransplanted into an adult patient in an autologous setting renders them an attractive cell source [7]. However, limitations include the production of mixed and immature cardiac cells [48]. To achieve a nodal-type specification, previous approaches used viral gene transduction of the pacemaker-related genes *HCN4*, *SHOX2*, or *TBX18* [41, 49, 50], leading to the risk of systemic infection and/or only transient effects of gene therapy.

We here report on the subtype-specific generation of cardiac pacemaker-type cells from hiPSC using a culture media-based approach. After 10–12 days of co-culture with END-2 cells [6] in serum-free medium, differentiated hiPSC lines (dhiPSC) formed beating clusters (~50 beats/min) and exhibited the abundant transcription of pacemaker hallmark genes. Action potential (AP) morphology at this early stage showed features highly similar to nodal-type APs, but slow rates and low amplitude indicated an immature nature to these cells (Additional files 10 and 11). Correspondingly, clusters expressed the working-type myocardial transcription factors Tbx5, Mef2c, and Nkx2.5, in addition to pacemaker-related genes, in line with a premature precursor cell type sharing nodal- and working-type cardiomyocyte properties [16–19]. Based on previous reports indicating that FBS increases the automaticity of pluripotent cell-derived cardiomyocytes [51], we evaluated whether timed transfer of dhiPSC from serum-free co-culture medium to a FBS-enriched medium immediately after beating onset may promote differentiation towards the nodal-type direction.

Clusters, cultured for 8 weeks according to our protocol, expressed high levels of pacemaker-related transcripts and proteins, in particular transcription factors (Tbx3, Tbx18, SHOX2), transporters (NCX1), connexins (Cx45), ion channels (HCN1, HCN4, Ca_v_1.2), and adrenergic receptors. At the same time, transcription factors (Tbx5, Mef2c, Nkx2.5), structural markers (cTnI, Cx40), and ion channels (Na_v_1.5, K_v_4.3, K_ir_2.1) related to working-type cardiomyocytes were downregulated. Nodal-type lineage specification was essentially indicated by an inverse relation of increasing firing rate and declining transcript levels of Nkx2.5 after exposure to FBS-enriched medium (Additional file 6A and C). In contrast, continued co-culturing in serum-free medium in a parallel culture resulted in robust Nkx2.5 levels and slowly declining rates (Additional file 6B and D), reflecting differentiation towards a primarily ventricular phenotype [6, 9]. Importantly, Nkx2.5 is a major regulator for sinus node development and nodal subtype specification. High Nkx2.5 transcription promotes differentiation into working-type cardiomyocytes [29], repressing SAN development [30]. Conversely, decline of Nkx2.5 is a prerequisite for activation of the pacemaker gene program [30]. Thus, downregulation of Nkx2.5 after a switch to the serum-enriched medium is considered as key to lineage specification in the pacemaker-type direction. This is underlined by the fact that culturing in serum-enriched medium leads to upregulation of SHOX2 (Fig. 2c), a transcription factor known to antagonize the transcriptional output of Nkx2.5 [52]. Moreover, the role of Nkx2.5 is further supported by a recent study demonstrating that pacemaker-type cells are recruited from Nkx2.5-negative progenitors [10].

Patch-clamp recordings revealed that a majority of cells (63.4%) isolated from PCC produced nodal-type APs. Interestingly, we observed a specified cellular arrangement within the clusters. While cells at the center formed elongated, spindle-shaped morphologies and exhibited spontaneous central SAN-type AP properties, more peripheral cells produced APs with a higher upstroke resembling atrial or peripheral SAN cells [43]. Of note, APs with plateau morphology, pointing to ventricular-type cardiomyocytes, were not observed. Furthermore, cells within PCC produce spontaneous Ca^2+^ transients characterized by regular repetitive oscillations in [Ca^2+^]_i_ pointing to a “coupled clock” mechanism that drives automaticity in pacemaker-type cells. To rule out the persistence of immature cells with retained proliferative activity and bearing a risk of tumorigenesis, we performed ATP-based proliferation assays prior and throughout differentiation. While proliferative activity was high in hiPSC, we found negligible proliferation of dhiPSC when cultured in the FBS-enriched medium for weeks.

Altogether, we reasoned that switching medium and subsequent long-term culture in the presence of FBS elicits subtype specification from immature cardiomyogenic precursors to progressively maturated nodal-type cells. This notion was further substantiated by functional assessment of PCC using MEA. Automaticity of PCC was rate responsive to adrenergic and cholinergic stimulation, indicating the capacity of clusters to modulate the rate according to autonomic input. In addition, treatment with ivabradine at a concentration that specifically inhibits the f-current (3 μM) [40] significantly reduced the beating rate, indicating a rate modulation facilitated by f-current, similar to the physiological situation in the SAN. In this regard, it was recently reported that ivabradine at relatively high concentrations of up to 9 μM did not alter the rate of spontaneously beating hiPSC-derived working-type cardiomyocytes [53], pointing to different mechanisms of automaticity in such cells. Baseline rates of PCC were regular and robust (70–90 beats/min) and persisted throughout long-term culture. Co-culture with NRVM indicated electrical integration of the PCC within the monolayer based on synchronous contraction of the whole culture at PCC baseline rates, which were significantly faster than NRVM monocultures.

To date, knowledge about medium-based lineage specification of pluripotent stem cells toward the nodal subtype is limited, and most data were produced in mouse embryonic stem cell lines (Additional file 12). Notably, these approaches beside the hanging drop method and diverse enrichment strategies usually apply a considerable amount of FBS (10–20%) as an integral component to achieve differentiation of attached cells. To investigate the subtype specification of SAN-like cells during differentiation, comparative expression analysis of SAN-specific marker genes *SHOX2* and *HCN4* have been established for positive control in these studies [52], according to our approach.

Future studies need to further explore the molecular mechanisms underlying co-culture- and serum-dependent cardiac lineage specification of human pluripotent cell lines. Moreover, the capability of PCC to induce in vivo biological pacemaker function needs to be investigated. In an experimental large animal model, however, such an approach may necessitate immunosuppression to enable transplantation of human-derived cells [19, 54] into an animal heart.

## Conclusion

We provide an efficient approach to differentiate stem cell-derived pacemaker-type cells that develop in clusters and possess the characteristic properties of nodal-type cardiomyocytes. Different from most previous strategies [55], our approach is based on human cells and does not require genetic manipulation to achieve pacemaker cell differentiation. Phenotypic features include not only electrical oscillation but also electrophysiological, cellular, and morphological properties of endogenous pacemaker cells. Our technology thus may facilitate in vitro studies of cellular mechanisms of sinus node disease using patient-specific iPSC and provides a platform for drug testing and for the generation of a biological pacemaker.

## Additional files

Additional file 1: Additional materials, methods, and references. (DOCX 23 kb)

Additional file 2: Teratoma formation of hiPSC. Teratomas formed after subcutaneous injection of hiPSC into the flanks of immunodeficient mice. Teratomas were stained positive for all three germ layers: ectodermal marker glial fibrillary acidic protein (GFAP) (A), endodermal marker pan-cytokeratin (B) and mesodermal marker vimentin (C). Scale bars = 100 μm. (TIF 5512 kb)

Additional file 3: Primers used for qRT-PCR. (DOC 895 kb)

Additional file 4: Antibodies used for immunocytochemical assays. (DOC 850 kb)

Additional file 5: Rate profiles of pacemaker cell clusters. Rate profiles of spontaneously beating pacemaker cell clusters (PCC) derived from three independent hiPSC lines over an observational period of 28 days (purple: hiPSC line #1; red: hiPSC line #2; black: hiPSC line #3). (TIF 181 kb)

Additional file 6: Spontaneous firing rate inversely correlates with Nkx2.5 transcription in pacemaker cell clusters. Spontaneous firing rate inversely correlates with Nkx2.5 transcription in PCC (A, C). In VCC, firing rates remain slow, consistent with robust high Nkx2.5 transcription (B, D). *PCC* pacemaker cell clusters, *VCC* ventricular-type cell clusters, *dhiPSC* co-cultured for 10 days, culture duration in weeks is denoted on the *x*-axis. (TIF 1182 kb)

Additional file 8: Movie of spontaneously beating PCC on MEA derived from hiPSC line #2. (AVI 2027 kb)

Additional file 9: Immunohistochemical analysis of pacemaker cell clusters. Signals were visualized by fluorescence microscopy. Left column: (A) anti-HCN1; (B) anti-HCN2; (C) left, anti-HCN4; (D) left, anti-Ca_v_1.2; (E) left, anti-Ca_v_1.3; (F) left, anti-Cx45. (A–F) Middle column: Nuclei of respective samples (A–F) are counterstained with DAPI. Right column: overlay of immunostains (A–F) and DAPI counterstain. Scale bars = 100 μm. (TIF 2774 kb)

Additional file 10: Representative recording of spontaneous APs of early differentiated iPSC-derived cells (dhiPSC). APs of dhiPSC (age 10 days) showed uniform morphologies most closely related to nodal-type APs, characterized by low amplitude, lack of overt plateau, depolarized maximal diastolic potential and a prominent and slow depolarization of the early phase of the AP. However, slow rates and low peak/amplitude indicate immature nature of cells. Please also refer to Additional file 11 for summary of AP data at day 10. (TIF 190 kb)

Additional file 11: Electrophysiological characterization of action potentials (APs) recorded from early differentiated iPSC-derived cells (dhiPSC, age 10 days) by current clamp technique. (DOC 29 kb)

Additional file 12: Overview of nodal lineage differentiation protocols in embryonic stem cells. (DOC 399 kb)

## Abbreviations

Amp: Amplitude
AP: Action potential
APD: Action potential duration
cTnI: Cardiac troponin I
Cx: Connexin
dhiPSC: Differentiated human induced pluripotent stem cell-derived clusters
dV/dt_max_: Maximal rate of depolarization
END-2: Visceral endoderm-like
FBS: Fetal bovine serum
HCN: Hyperpolarization-activated cyclic nucleotide channels
hESC: Human embryonic stem cells
hiPSC: Human induced pluripotent stem cells
hRA: Human right atrium
hSAN: Human sinoatrial node
ISO: Isoproterenol
IVA: Ivabradine
MDP: Maximum diastolic potential
MEA: Multi-electrode array
MEF: Mouse embryonic fibroblasts
NCX1: Sodium calcium exchanger 1
NRVM: Neonatal rat ventricular myocytes
PBS: Phosphate-buffered saline
PCC: Pacemaker cell cluster
qRT-PCR: Quantitative real-time polymerase chain reaction
SAN: Sinoatrial node
SSS: Sick sinus syndrome
