# Supplementary material for: Subtype-specific differentiation of cardiac pacemaker cell clusters from human induced pluripotent stem cells
Source: Stem Cell Res Ther. 2017 Oct 16;8:229. doi: 10.1186/s13287-017-0681-4 (PMC5644063; doi:10.1186/s13287-017-0681-4)
Supplement: Supplementary file 2 — Additional materials, methods, and references. (DOCX 23 kb) [file 13287_2017_681_MOESM1_ESM.docx]

**Additional file 1**

**Subtype-specific differentiation of cardiac pacemaker cell clusters from human induced pluripotent stem cells**

Patrick A. Schweizer*, Fabrice F. Darche, Nina D. Ullrich, Pascal Geschwill, Boris Greber, Rasmus Rivinius, Claudia Seyler, Karin Müller-Decker, Andreas Draguhn, Jochen Utikal, Michael Koenen, Hugo A. Katus, Dierk Thomas

* Corresponding author. E-mail: patrick.schweizer@med.uni-heidelberg.de

**ADDITIONAL MATERIALS AND METHODS**

**Generation of hiPSC line #1**

8x10^4^ human dermal fibroblasts (HDF, BioCat GmbH, Heidelberg, Germany, http://www.biocat.com) were seeded on 6-well-plates (Greiner Bio-One International AG, Kremsmünster, Austria, http://www.gbo.com) coated with 0.1% gelatin (Sigma-Aldrich, St. Louis, MO, http://www.sigmaaldrich.com) /phosphate buffered saline (PBS, Thermo Fisher Scientific, Inc., Waltham, MA, http://www.thermoscientific.com)-solution. HDF were cultured in 2 ml HDF medium containing Dulbecco’s Modified Eagle Medium (DMEM, Thermo Fisher Scientific), 10% FBS (FBS, Thermo Fisher Scientific), 100 U/ml penicillin (Thermo Fisher Scientific) and 100 µg/ml streptomycin (Thermo Fisher Scientific). The following day, HDF were infected with Oct4, Sox2, Klf4 and cMyc lentiviral particles (all from Allele Biotechnology, San Diego, CA, http://www.allelebiotech.com). A multiplicity of infection (MOI) of 10 was used. 24 hours later, HDF medium was added and medium change with 2 ml fresh HDF medium was performed 48 hours after infection of the cells.

10^6^ mitomycin C treated mouse embryonic fibroblasts (MEF, BioCat GmbH, Heidelberg, Germany, # CBA-310-CB) were seeded on a 6-well plate coated with 0.1% gelatin/PBS-solution. Mitomycin C treated MEF served as feeder cells for the maintenance of pluripotency of hiPSC. MEF feeder cells were cultured in MEF medium containing DMEM, 10% FBS, 100 U/ml penicillin and 100 µg/ml streptomycin. 72 hours after infection, HDF were transferred on MEF feeder cells and cultured in hiPSC medium containing Knockout DMEM (Thermo Fisher Scientific), 20 % Knockout serum replacement (KOSR, Thermo Fisher Scientific), 100 µM ascorbic acid (Sigma-Aldrich), 100 U/ml penicillin, 100 µg/ml streptomycin, 10 µg/ml bFGF (PeproTech, Rocky Hill, NJ, http://www.peprotech.com) and 10^-4^ M 2-mercaptoethanol (Sigma-Aldrich).

Flat, sharply delineated hiPSC colonies emerged 3 weeks after HDF infection. They were mechanically detached from the MEF feeder cells under light microscopy (microscope Wilovert, Helmut Hund GmbH, Wetzlar, Germany, http://www.hund.de) and were transferred on a 6-well-plate with fresh MEF feeder cells. Medium change was performed daily. HiPSC colonies were passaged when borders of two colonies adjoined. To do so, cell passaging was performed by adding 1 ml of 1 mg/ml collagenase IV (Thermo Fisher Scientific)/PBS-solution per well. After incubation of hiPSC with collagenase IV-solution for 5 min at 37°C, 1 ml of hiPSC culture medium was added to each well, followed by a wash in phosphate buffered saline solution. HiPSC colonies were mechanically dissociated, transferred to a 50 ml Falcon vial (BD Biosciences, San José, CA, http://www.bdbiosciences.com) containing 10 ml hiPSC medium supplemented with 10 µM Y-27632 dihydrochloride (Abcam, Cambridge, UK, http://www.abcam.com). Cells were then centrifuged for 1 min at 800 rpm. Subsequently, cell supernatant was discarded and the cell pellet was resuspended in 3 ml of hiPSC medium supplemented with 10 mM Y-27632. For hiPSC expansion 1 ml of hiPSC suspension was transferred into a 50 ml Falcon vial containing 23 ml of hiPSC medium supplemented with 10 µM Y-27632 dihydrochloride and hiPSC colony fragments were distributed on two 6-well-plates with MEF feeder cells. HiPSC medium was changed daily. Freezing of hiPSC : an equal amount of freezing medium consisting of 80 % serum replacement and 20 % dimethylsulfoxide (DMSO, Merck) was added to the remaining 2 ml of hiPSC cell suspension. HiPSC cell suspension was distributed to 4 cryovials (Greiner), which were stored in a nitrogene tank at -196 °C.

**Generation of hiPSC line #2**

The iPSC line FS3F.2 has been described in Greber et al. [1]. Briefly, this line was produced from human foreskin fibroblasts (ATCC #CRL-2097) using Melton's protocol [2] with 3 factors (OCT4, SOX2, KLF4). Retroviruses were produced in 293T cells using Fugene 6 (Roche) and Addgene plasmids 8454, 8449, 17217, 17218, and 17219 [3]. Line FS3F.2 has been fully characterized according to standard procedures [1].

**Generation of hiPSC line #3**

hiPSC line #3 was generated from primary human fibroblasts derived from a skin biopsy of a healthy donor using a doxycycline-inducible polycistronic lentiviral vector (STEMCCA-OKSM) and a tet-activator M2rtTA (Ethics Committee of Heidelberg University approval no. 2009-350N-MA). For hiPSC generation, primary human fibroblasts were seeded at a density of 5000 cells/well on gelatin coated 6-well plates in DMEM containing 10% FBS, 1x non-essential amino acids, 1% penicillin/streptomycin and 50 µM ß-mercaptoethanol (fibroblast cell culture medium). For lentiviral transduction, 5 µL of M2rtTA and 5 µL STEMCCA-OKSM concentrated viral particles were added. The following day, cells were rinsed with PBS followed by another round of lentiviral transduction. After 24 h the cells were washed twice with PBS and a 1:1 mixture of fibroblast cell culture medium with human ES medium (DMEM/F12 (Thermo Fisher Scientific) supplemented with 20 % KOSR, 1% L-glutamine, 1% penicilline/streptomycine, 1% MEM-NEAA, 10^-4^ M 2-Mercaptoethanol and 10 ng/ml bFGF) was added to the cells. In order to induce the transgene expression the medium was supplemented with 1 µg/mL doxycycline (Sigma-Aldrich). The transduced cells were cultured in the 1:1 mixture medium for one week, afterwards in hES medium supplemented with 1 µg/mL doxycycline. After three to five weeks appearing colonies of reprogrammed cells were picked manually and transferred onto mitomycin c (Sigma-Aldrich) -treated mouse embryonic feeder cells in hES medium. After another two weeks, when the cells reached a stable state, doxycycline was withdrawn and the colonies were further expanded using standard methods for human pluripotent stem cells.

***In vivo* teratoma formation in mice**

We performed teratoma assays with hiPSC lines #1-3. Female Scid beige mice (Charles River Laboratories, Wilmington, MA) were maintained in the central animal facility of the German Cancer Research Center, Heidelberg at a 12 h light–dark cycle with unrestricted Kliba Nafag diet 3307 (Provimi Kliba AG, Kaiseraugst, Switzerland, http://www.kliba-nafag.ch) and water. Animal experimentation was done in accordance with regional regulatory authorities (reference number G-158/11). At the age of 7 weeks, mice (n = 5/group) were injected under isoflurane anesthesia (1 - 1.5 % in O2, 0.5 l/min) subcutaneously with about 1x106 cells in a 1:1 mixture of 100 µl PBS/Matrigel (BD Biosciences) into the right flank. Growth of subcutaneous tumors was monitored and xenografts were dissected when reaching 1.5 cm in one dimension, i. e. about 6 weeks after injection. For RNA and protein isolation, half of the tumor was snap-frozen in liquid nitrogen. For histological and immunohistochemical analysis, a quarter of the tumor was fixed in 4 % PBS-buffered paraformaldehyde and paraffin-embedded and one quarter was cryopreserved in TissueTek OCT compound.

**Immunohistochemistry**

Routine hematoxylin and eosin stainings as well as immunohistochemistry were performed on 5 µm paraffin sections as described previously [4]. To stain the teratomas for ectodermal (GFAP), mesodermal (vimentin) and endodermal (pan cytokeratin) markers antibodies given in additional Table S2 were used. Pictures were taken by means of an Axioskop 2 microscope equipped with an Axiocam and Axiovision software (Carl Zeiss AG, Oberkochen, Germany, http://www.zeiss.de).

**ADDITIONAL REFERENCES**

1. Greber B, Coulon P, Zhang M et al. FGF signalling inhibits neural induction in human embryonic stem cells. EMBO J 2011;30:4874-4884.
2. Huangfu D, Maehr R, Guo W et al. Induction of pluripotent stem cells by defined factors is greatly improved by small-molecule compounds. Nat Biotechnol 2008;26: 795-797.
3. Takahashi K, Tanabe K, Ohnuki M et al. Induction of pluripotent stem cells from adult human fibroblasts by defined factors. Cell 2007;131:861-872.
4. Vegiopoulos A, Müller-Decker K, Strzoda D et al. Cyclooxygenase-2 controls energy homeostasis in mice by de novo recruitment of brown adipocytes. Science 2010;328: 1158-1161.
