# Supplementary figures and images for: Subtype-specific differentiation of cardiac pacemaker cell clusters from human induced pluripotent stem cells
Source: Stem Cell Res Ther. 2017 Oct 16;8:229. doi: 10.1186/s13287-017-0681-4 (PMC5644063; doi:10.1186/s13287-017-0681-4)

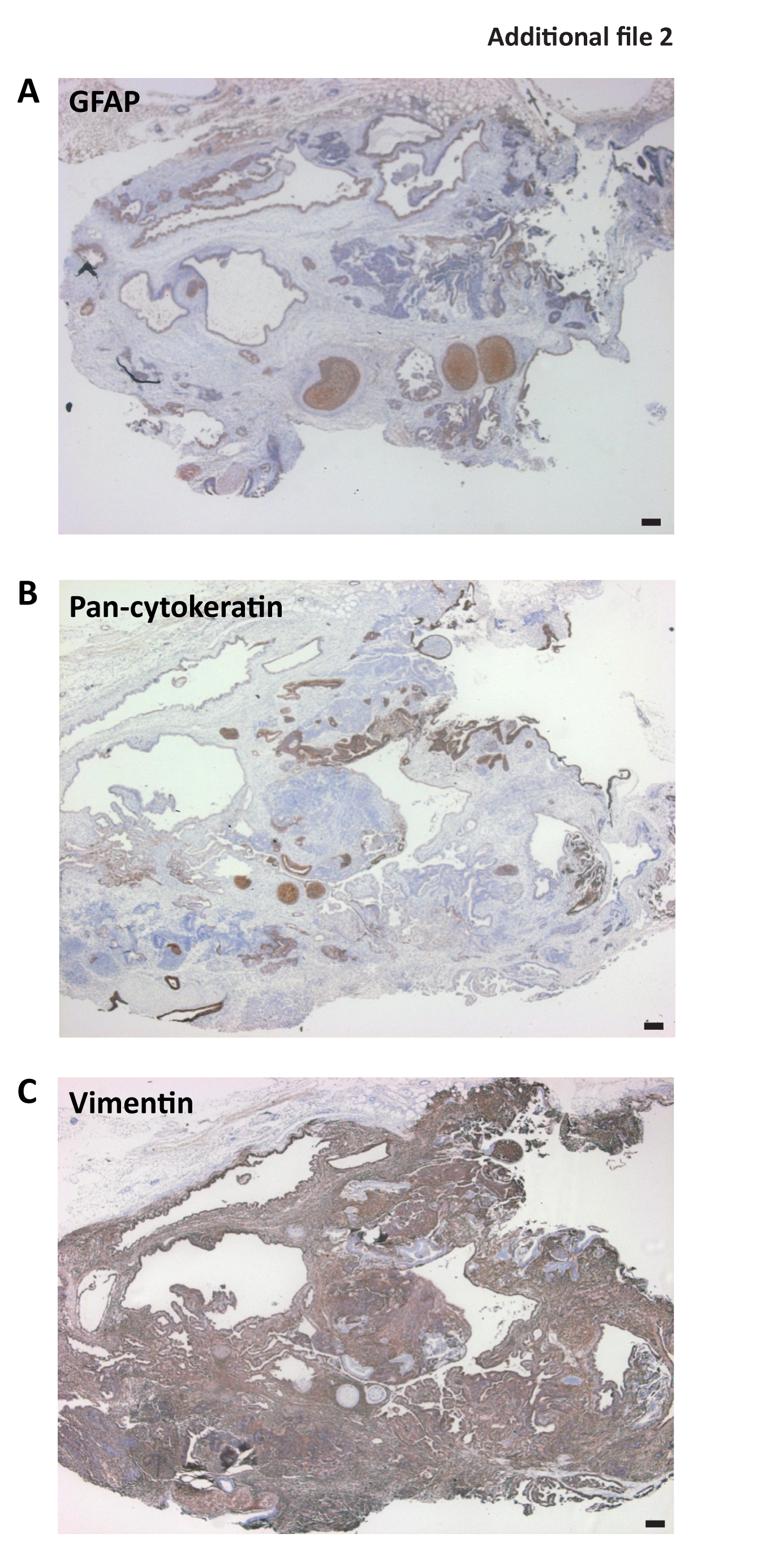

Supplement: Supplementary file 3 — Teratoma formation of hiPSC. Teratomas formed after subcutaneous injection of hiPSC into the flanks of immunodeficient mice. Teratomas were stained positive for all three germ layers: ectodermal marker glial fibrillary acidic protein (GFAP) (A), endodermal marker pan-cytokeratin (B) and mesodermal marker vimentin (C). Scale bars = 100 μm. (TIF 5512 kb) [file 13287_2017_681_MOESM2_ESM.tif]

**Additional file 3.** Primers used for quantitative real-time polymerase chain reaction (qRT-PCR)


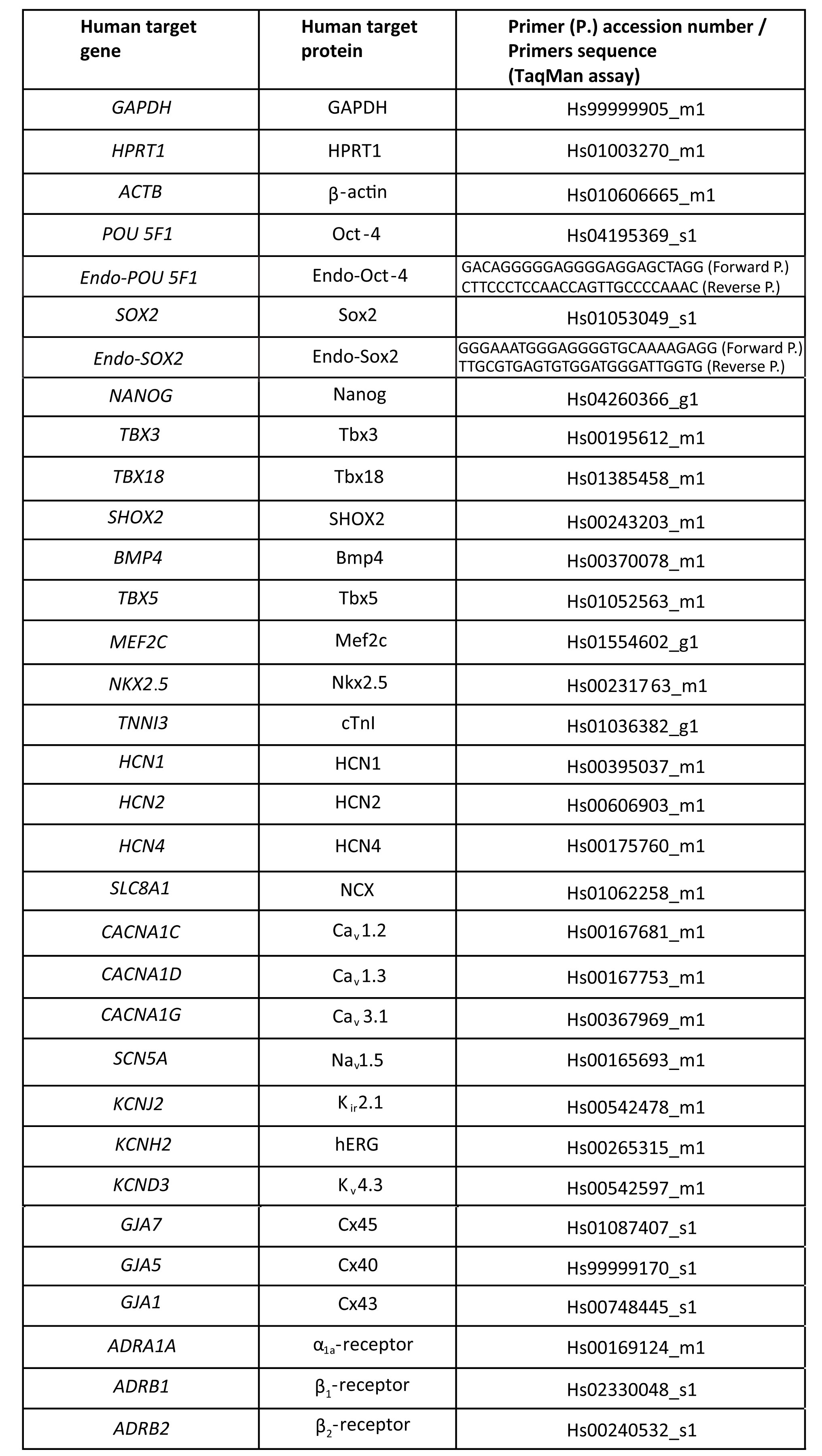

Supplement: Supplementary file 4 — Primers used for qRT-PCR. (DOC 895 kb) [file 13287_2017_681_MOESM3_ESM.doc]

**Additional file 4.** Antibodies used for immunocytochemical assays.


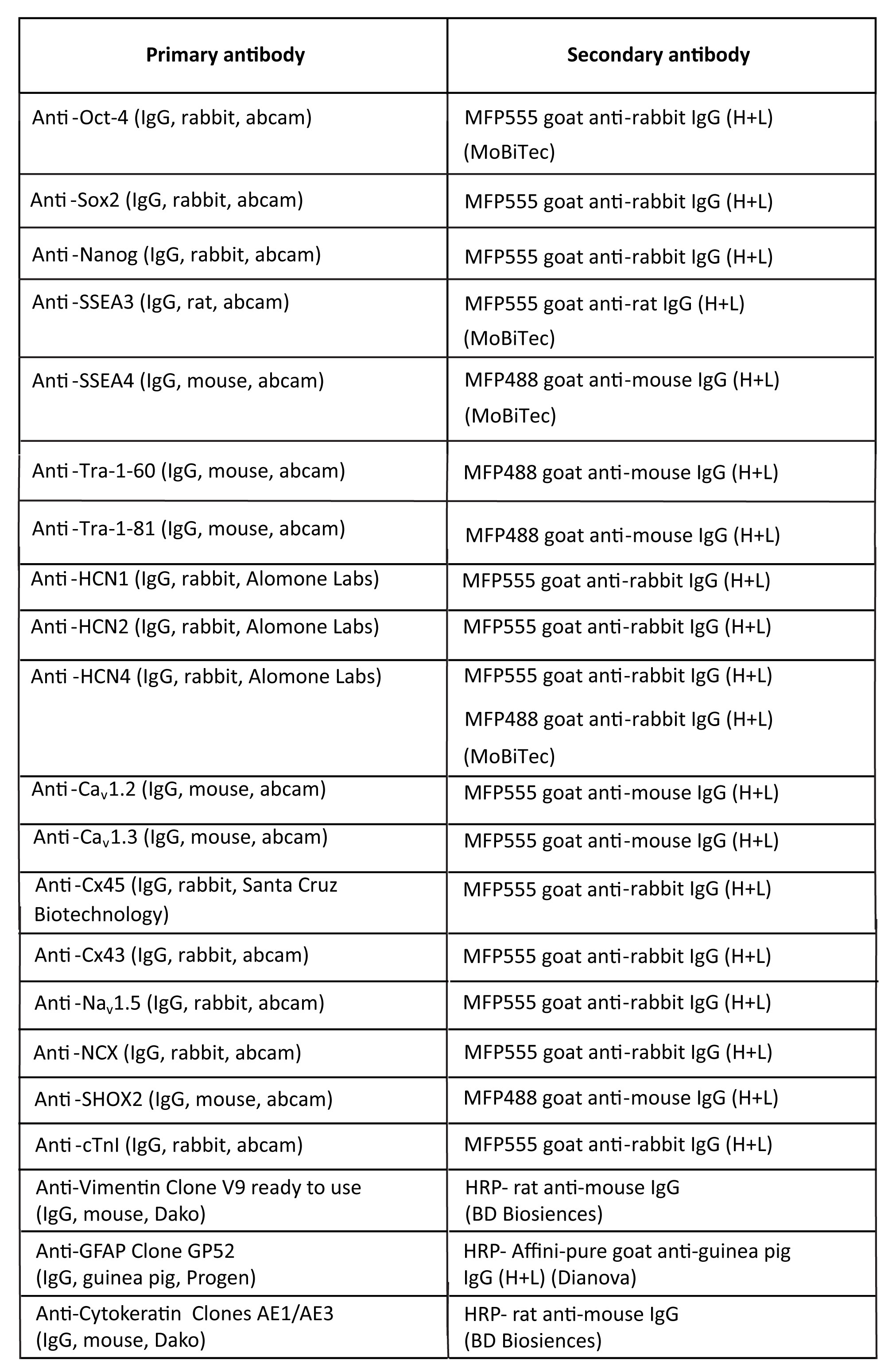

Supplement: Supplementary file 5 — Antibodies used for immunocytochemical assays. (DOC 850 kb) [file 13287_2017_681_MOESM4_ESM.doc]

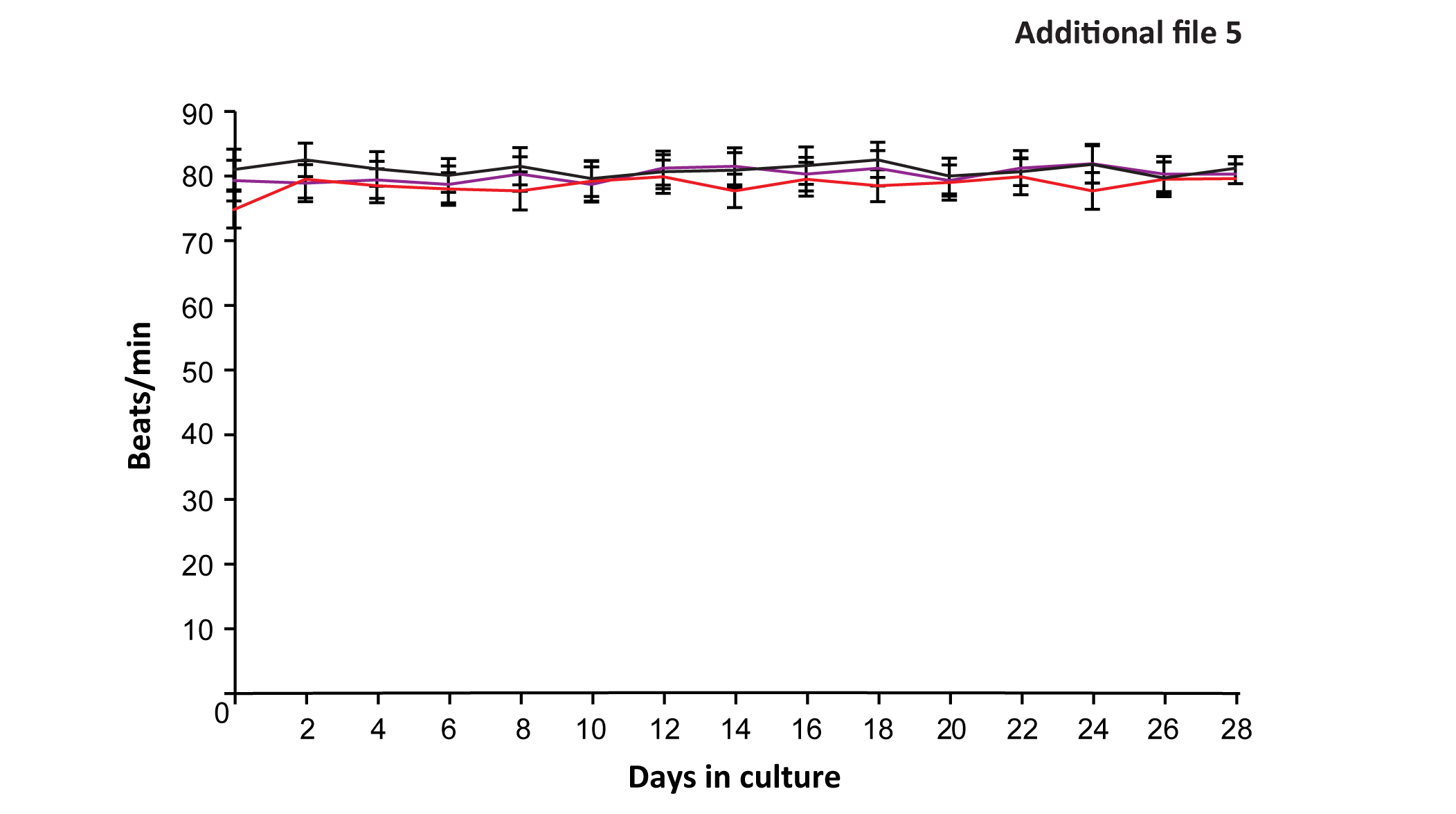

Supplement: Supplementary file 6 — Rate profiles of pacemaker cell clusters. Rate profiles of spontaneously beating pacemaker cell clusters (PCC) derived from three independent hiPSC lines over an observational period of 28 days (purple: hiPSC line #1; red: hiPSC line #2; black: hiPSC line #3). (TIF 181 kb) [file 13287_2017_681_MOESM5_ESM.tif]

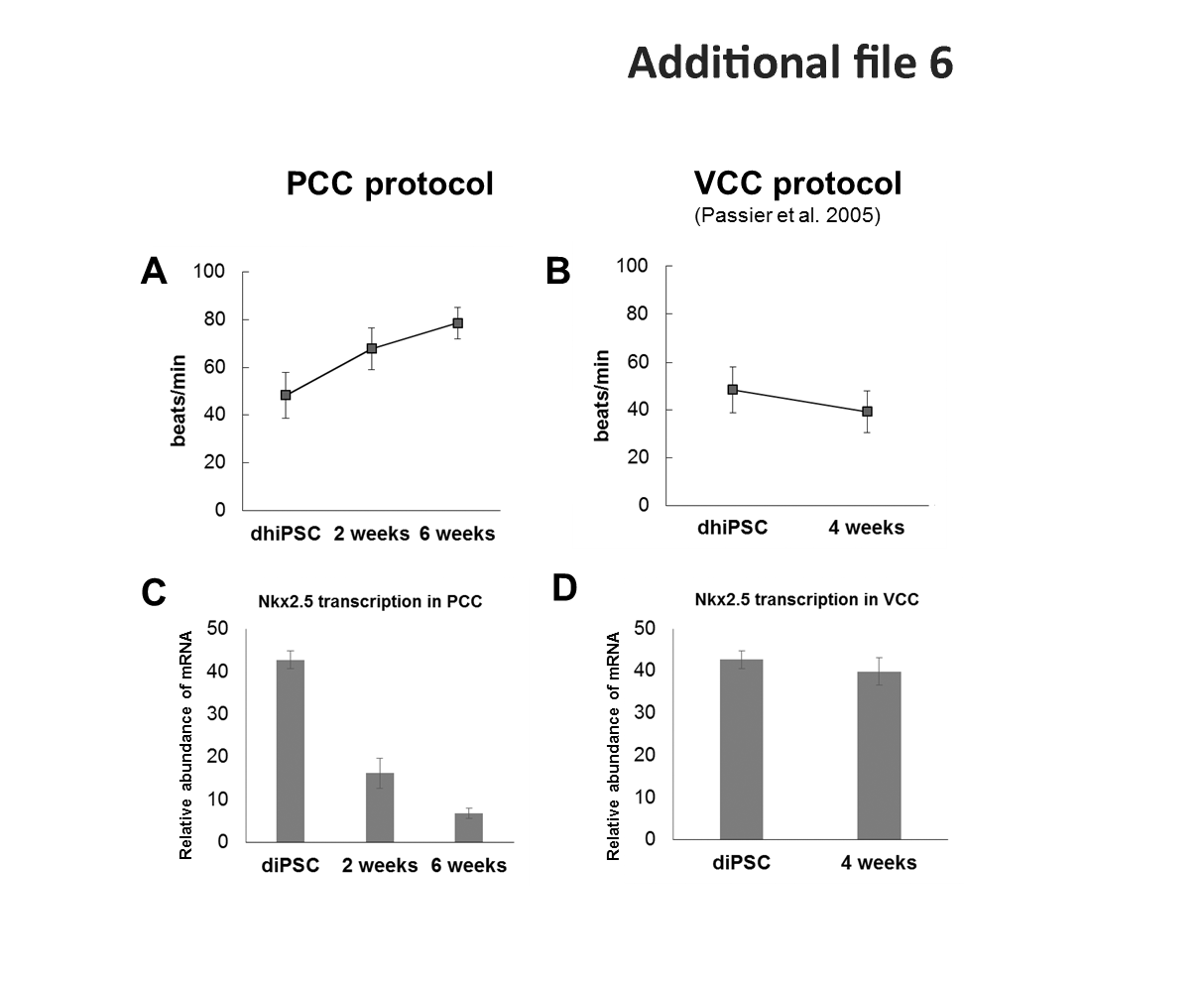

Supplement: Supplementary file 7 — Spontaneous firing rate inversely correlates with Nkx2.5 transcription in pacemaker cell clusters. Spontaneous firing rate inversely correlates with Nkx2.5 transcription in PCC (A, C). In VCC, firing rates remain slow, consistent with robust high Nkx2.5 transcription (B, D). PCC pacemaker cell clusters, VCC ventricular-type cell clusters, dhiPSC co-cultured for 10 days, culture duration in weeks is denoted on the x-axis. (TIF 1182 kb) [file 13287_2017_681_MOESM6_ESM.tif]

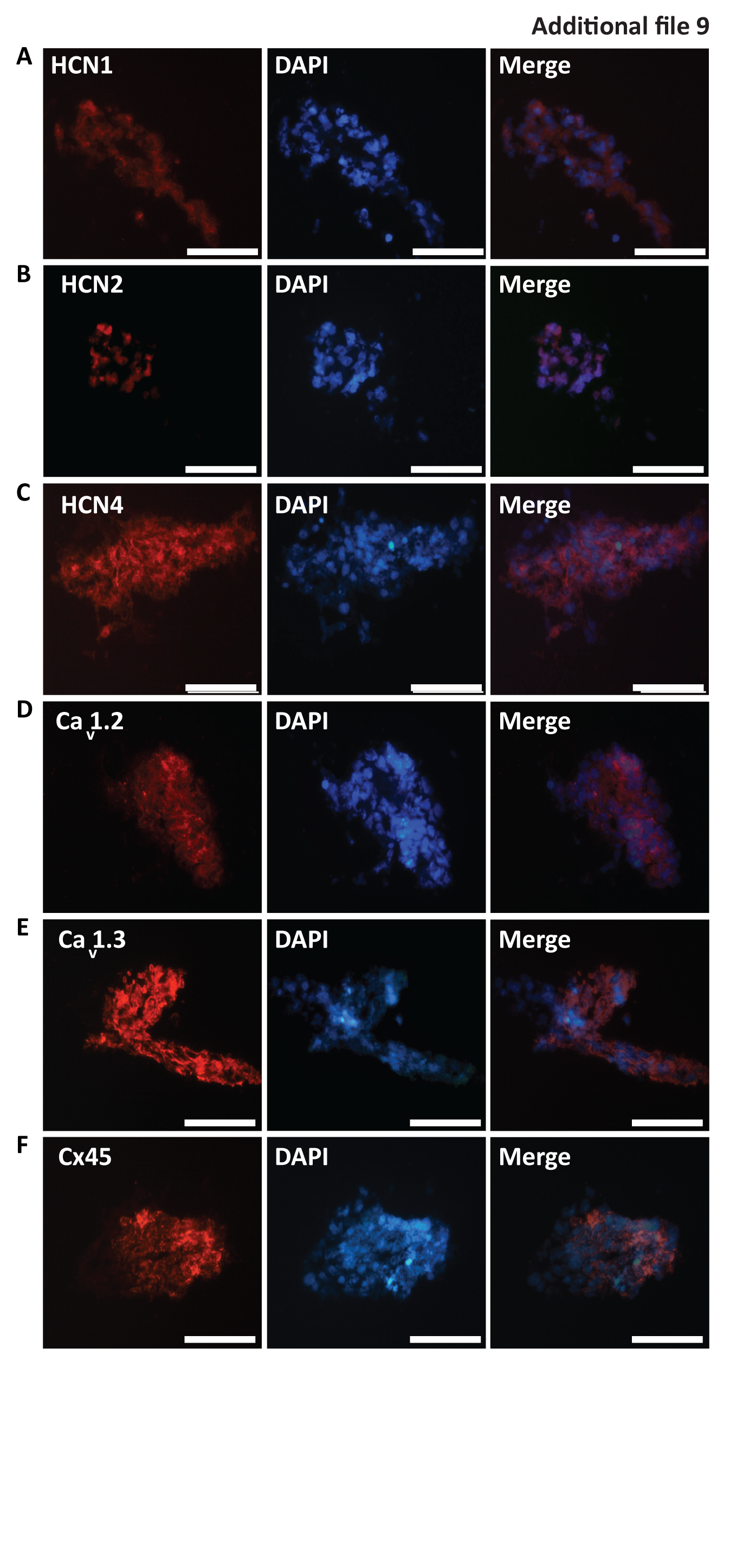

Supplement: Supplementary file 9 — Immunohistochemical analysis of pacemaker cell clusters. Signals were visualized by fluorescence microscopy. Left column: (A) anti-HCN1; (B) anti-HCN2; (C) left, anti-HCN4; (D) left, anti-Cav1.2; (E) left, anti-Cav1.3; (F) left, anti-Cx45. (A–F) Middle column: Nuclei of respective samples (A–F) are counterstained with DAPI. Right column: overlay of immunostains (A–F) and DAPI counterstain. Scale bars = 100 μm. (TIF 2774 kb) [file 13287_2017_681_MOESM9_ESM.tif]

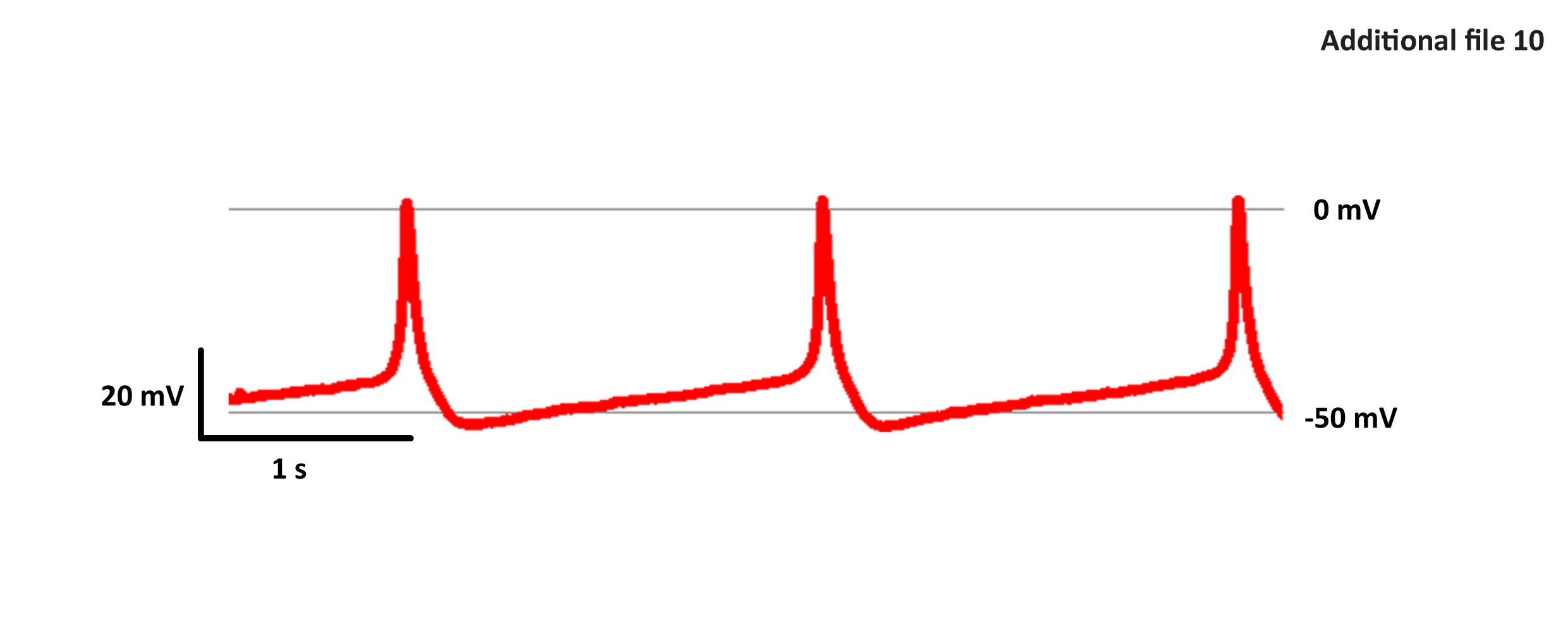

Supplement: Supplementary file 10 — Representative recording of spontaneous APs of early differentiated iPSC-derived cells (dhiPSC). APs of dhiPSC (age 10 days) showed uniform morphologies most closely related to nodal-type APs, characterized by low amplitude, lack of overt plateau, depolarized maximal diastolic potential and a prominent and slow depolarization of the early phase of the AP. However, slow rates and low peak/amplitude indicate immature nature of cells. Please also refer to Additional file 11 for summary of AP data at day 10. (TIF 190 kb) [file 13287_2017_681_MOESM10_ESM.tif]

**Additional file 12.** Summary of nodal lineage differentiation protocols in embryonic stem cells.


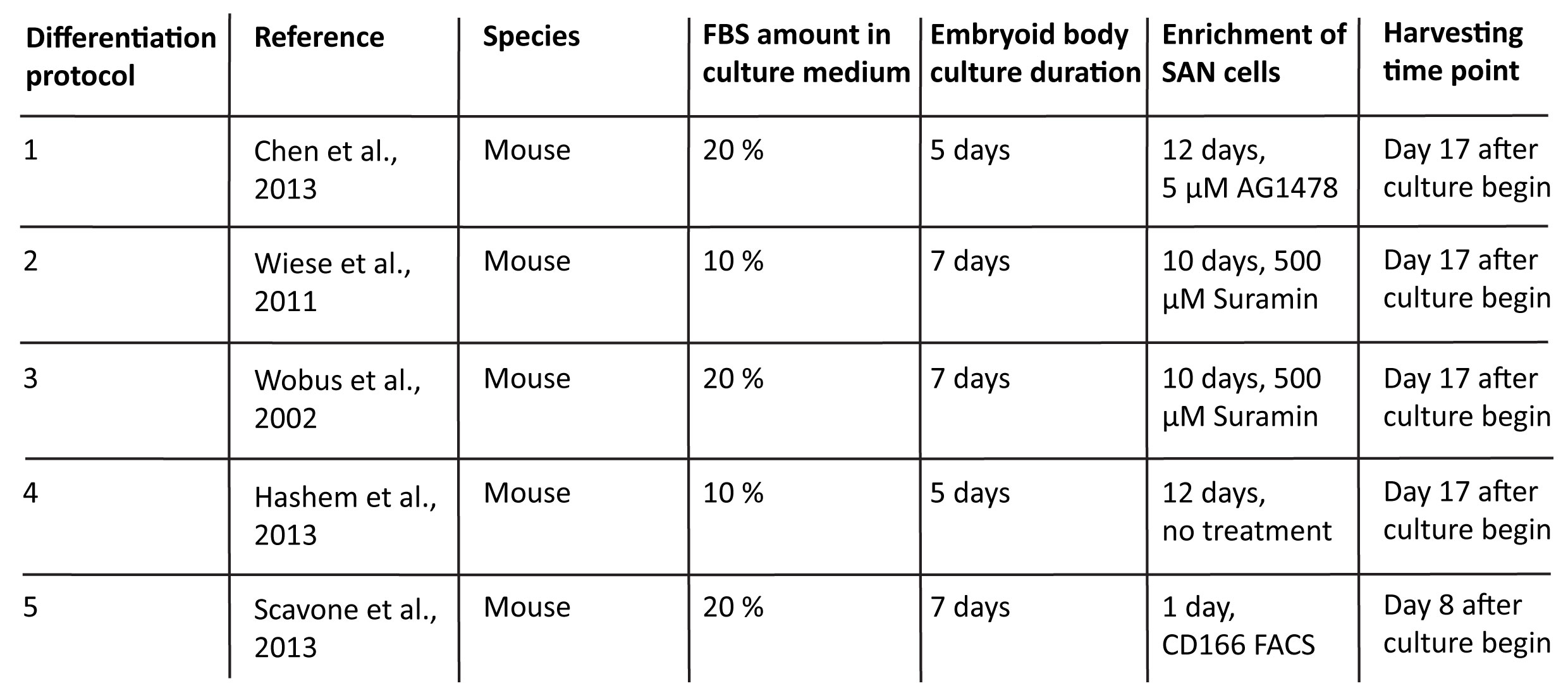

Supplement: Supplementary file 12 — Overview of nodal lineage differentiation protocols in embryonic stem cells. (DOC 399 kb) [file 13287_2017_681_MOESM12_ESM.doc]
