# Supplementary material for: Subtype-specific differentiation of cardiac pacemaker cell clusters from human induced pluripotent stem cells
Source: Stem Cell Res Ther. 2017 Oct 16;8:229. doi: 10.1186/s13287-017-0681-4 (PMC5644063; doi:10.1186/s13287-017-0681-4)
Supplement: Supplementary file 11 — Electrophysiological characterization of action potentials (APs) recorded from early differentiated iPSC-derived cells (dhiPSC, age 10 days) by current clamp technique. (DOC 29 kb) [file 13287_2017_681_MOESM11_ESM.doc]

**Additional file 11.** Electrophysiological characterization of action potentials (APs) recorded from early differentiated iPSC-derived cells (dhiPSC, age 10 days) by current clamp technique.

| **APs** | **MDP** | **Peak** | **Amp** | **dU/dt_max_** | **APD_30_** | **APD_40_** | **APD_70_** | **APD_80_** |
| --- | --- | --- | --- | --- | --- | --- | --- | --- |
|  | **[mV]** | **[mV]** | **[mV]** | **[V/s]** | **[ms]** | **[ms]** | **[ms]** | **[ms]** |
| **Mean** | **-49.20** | **-3.68** | **45.52** | **0.78** | **136.62** | **175.12** | **309.46** | **474.18** |
| **SEM** | **1.75** | **8.16** | **9.33** | **0.31** | **31.21** | **35.78** | **45.83** | **50.42** |

Maximum diastolic potential (MDP), peak voltage (Peak), amplitude (Amp), maximal rate of depolarization (dV/dt_max_), AP duration (APD) at different levels of repolarization. Measurements from 5 different cells are shown.
